# Potential Distribution Modeling and Conservation Gap Identification for Rare and Endangered Plant Species: A Case Study of 10 Species in Hubei Province

**DOI:** 10.1002/ece3.72672

**Published:** 2025-12-18

**Authors:** Dongyi Li, Tingting Li, Juyang Wu, Meng Zhang, Zhengxiang Wang

**Affiliations:** ^1^ Faculty of Resources and Environmental Science Hubei University Wuhan China; ^2^ Hubei Key Laboratory of Regional Development and Environmental Response, Faculty of Resources and Environmental Science Hubei University Wuhan China

**Keywords:** arbon storage, gap, habitat quality, Hubei Province, rare and endangered plant species, SDMs‐InVEST

## Abstract

With the continuous increase in the demand for land and natural resources, driven by population growth and economic expansion, the conservation of rare and endangered species faces mounting pressure. Exploring how to achieve the target of conserving 30% of the global terrestrial area proposed by the Kunming–Montreal Global Biodiversity Framework is of great significance for biodiversity and species protection. This study employed a combined SDMs–InVEST modeling approach to predict the current and future potential distributions, habitat quality, and carbon storage of 10 protected plant species in Hubei Province. Using the high‐potential distribution areas of these 10 species as conservation targets, combined with regions of high habitat quality and carbon storage, Marxan was applied to identify conservation gaps for these 10 protected plant species in Hubei Province. Results indicate that the province‐wide mean Habitat Quality Index (HQI) is projected to increase gradually from 0.355 to 0.366, while spatial heterogeneity of HQI will become more pronounced—western mountainous areas show marked HQI improvements, whereas HQI around central–eastern urban agglomerations declines significantly. Total ecosystem carbon storage in Hubei is projected to rise from 2.11 × 10^9^ t to 2.13 × 10^9^ t. On the basis of 423 occurrence records spanning 10 species (10 genera, 9 families), ensemble SDMs found climate to be the primary determinant of potential distributions; however, the future influence of anthropogenic disturbance and effects of habitat patches (EHPs) is projected to increase, leading to a 2.6% contraction in the total area of core potential distribution zones. These findings provide spatially explicit scientific guidance for optimizing regional protected‐area networks and for aligning biodiversity conservation with carbon management objectives under China's dual‐carbon strategy. Furthermore, multi‐period systematic conservation planning revealed significant protection gaps in interprovincial mountainous regions, forming four key aggregation zones: the Jinqian River source region (Shiyan–Shaanxi), the Tongbai Mountain belt (Suizhou–Henan), the Mufu Mountain region (Xianning), and the Wuling Mountain corridor (Enshi–Chongqing). These regions represent future conservation priorities. Our findings indicate that priority should be given to establishing new nature reserves selected from the 219 conservation gap planning units identified in this study, in order to strengthen the regional conservation‐planning system for rare and endangered plants in Hubei Province and to provide scientific and theoretical support for achieving the targets of the Kunming–Montreal Global Biodiversity Framework.

## Graphical abstract

An integrated SDMs‐InVEST approach coupled with SPP and HPP was applied to quantify and identify conservation gaps in Hubei Province. Although habitat quality is projected to improve, spatial heterogeneity and landscape fragmentation will intensify across the region. Multi‐period Marxan simulations revealed four major conservation gap clusters in interprovincial mountainous regions of Hubei Province.

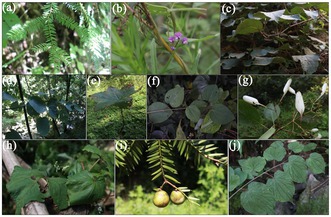

## 1 Introduction

Biodiversity is a critical component of the Earth's ecosystems, not only providing the material foundation necessary for human survival but also supporting essential ecosystem services and resilience through intricate interspecies interactions. However, over the past century, biodiversity loss driven by human activities has occurred at rates 30 to 120 times higher than the background extinction rates recorded in the Cenozoic fossil record (Senior et al. 2024). While the causes are multifaceted, the root driver lies in the surging demand for land and natural resources, spurred by population growth and economic expansion, which has led to widespread habitat conversion and degradation (Marques et al. 2019). Future biodiversity trajectories will increasingly diverge under different combinations of climate change and land‐use patterns within the framework of Shared Socioeconomic Pathways (SSPs), making scenario‐based conservation planning essential (Pang et al. 2024). In response, China has proposed systematic conservation strategies grounded in its dual‐carbon goals, such as the construction of an ecological civilization and a protected area system centered on national parks, which are expected to be more conducive to biodiversity conservation (Yang et al. 2019). The Kunming–Montreal Global Biodiversity Framework calls for protecting at least 30% of global terrestrial and marine areas by 2030 through protected areas and other effective area‐based conservation measures (OECMs), with particular attention to areas of high biodiversity value and human benefit (UNEP‐WCMC, IUCN, NGS 2020). Many of these biodiversity‐friendly actions also offer synergistic benefits for climate mitigation (Shin et al. 2022).

**FIGURE 1:**
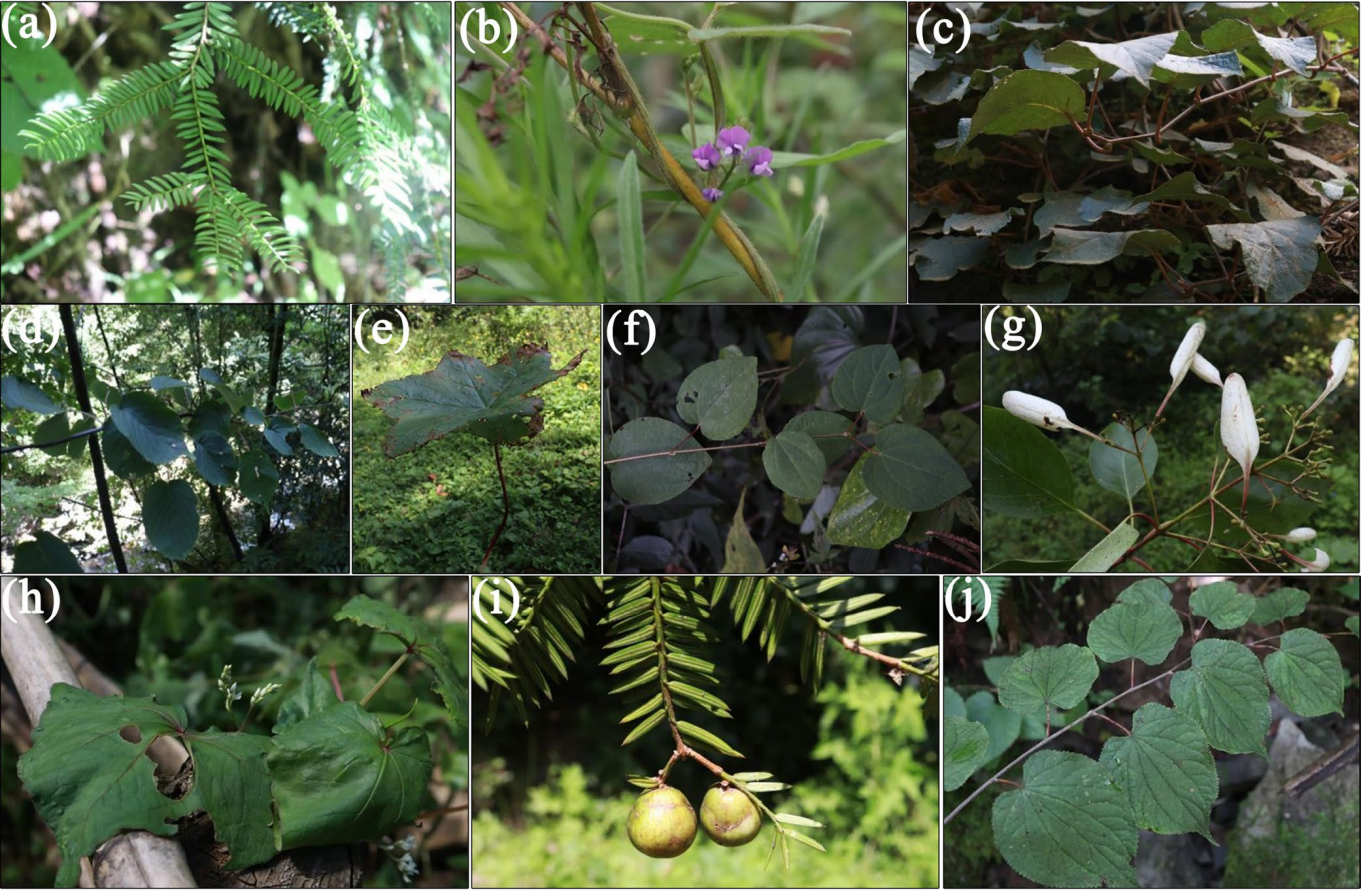
Organism photograph (Panels a–j show wild individuals photographed in the field of *Taxus wallichiana* var. *chinensis*, *Glycine soja* , *Actinidia chinensis* , *Davidia involucrata* var. *vilmoriniana*, *Dysosma versipellis*, *Cercidiphyllum japonicum* , *Emmenopterys henryi*, *Fagopyrum dibotrys*, *Torreya fargesii*, and *Tetracentron sinense*, respectively).

Protecting forests and rare, endangered plant species is vital to maintaining ecosystem integrity and biodiversity (Ralimanana et al. 2022). Compared to the conservation of endangered birds or reptiles and amphibians, the protection of endangered plant species offers a broader umbrella effect, benefiting multiple taxonomic groups (Dobson et al. 1997). This study investigates the suitable distribution of rare and endangered plants from various evolutionary branches—gymnosperms such as *Taxus wallichiana* var. *chinensis* and *Torreya fargesii*; ancient angiosperms such as *Cercidiphyllum japonicum* and *Tetracentron sinense*; and modern angiosperms such as *Davidia involucrata* var. *vilmoriniana*. Special focus is given to *Taxus wallichiana* var. *chinensis*, a glacial relict species from the Quaternary period, to understand the mechanisms that allowed its persistence amid long‐term climate fluctuations and geological changes—an insight of critical value for future climate adaptation strategies (Qi et al. 2012). In addition, *Glycine soja* , *Actinidia chinensis* , and *Fagopyrum dibotrys*—wild relatives of key Chinese crops and valuable genetic reservoirs—are also examined in the context of climate change and anthropogenic pressures, offering implications for food security and agricultural climate resilience (Meilleur and Hodgkin 2004). Hubei Province, one of China's key biodiversity hotspots, spans both northern and central subtropical zones and features diverse topography, creating a mosaic of habitats suitable for a wide range of rare and endangered species. Field investigations have identified vertical plant zonation in the Qinling‐Daba mountain remnants in northwestern Hubei, where *Taxus wallichiana* var. *chinensis*, *Torreya fargesii*, and *Davidia involucrata* var. *vilmoriniana* coexist. In the Wuling Mountains of southwestern Hubei, *Cercidiphyllum japonicum* and *Tetracentron sinense* form community dominants in mixed evergreen‐deciduous forests. Meanwhile, generalist species like *Glycine soja* reproduce naturally in ecotones between the Jianghan Plain and adjacent mountains, but their populations are being diminished by agricultural encroachment (Yang et al. 2019). Therefore, future conservation studies of these species will become increasingly important (Figure 1). Constructing an Ecological Security Pattern (ESP) focused on habitat quality is crucial for maintaining biodiversity, as habitat integrity underpins effective ESP design (Luedtke et al. 2023; Olds et al. 2014). Xu and Li (2025) emphasizes the importance of integrated approaches that consider both species and habitat perspectives for future conservation. Global studies spanning multiple taxa, spatial scales, and years have shown that habitat fragmentation can reduce biodiversity by 13%–75%, disrupt ecological function by decreasing biomass and altering nutrient cycles, and exacerbate extinction risks, especially in small and isolated habitat patches (Haddad et al. 2015). Additionally, the degree of habitat loss can induce a hump‐shaped biodiversity response to fragmentation, potentially accelerating species loss (Zhang, Chase, and Liao 2024). Most existing studies focus on single reserves, leaving the relationship between habitat fragmentation and biodiversity at broader regional scales poorly understood (Yuan et al. 2024).

Although the global expansion of protected areas (PAs) has played a pivotal role in maintaining biodiversity (Watson et al. 2014), most PAs in China are still designed based on expert opinion and short‐term ecological data. These limitations hinder their capacity to reflect long‐term ecological dynamics such as species migration and succession, thereby contributing to persistent protection gaps (Bull et al. 2013; Li and Pimm 2020). Future conservation strategies must integrate spatially explicit data on species, habitat, and human pressures using robust modeling tools to identify gaps and optimize PA networks.

Research on biodiversity conservation gaps has become a leading focus in ecological science. Studies using MaxEnt to map potential bird distributions have identified key gaps and informed nature reserve design using bird diversity as an indicator group (Xu and Li 2025; Yang et al. 2025). However, single‐model predictions often suffer from over‐ or underestimation due to limitations in accuracy and generalizability. Ensemble models, by integrating outputs from multiple algorithms, can provide more robust predictions and better inform conservation planning (Street 2020). Zhang et al. (2022), for instance, evaluated habitat quality as a cost parameter in Marxan‐based planning to prioritize conservation areas in the Beijing–Tianjin–Hebei region. Such work underscores the importance of baseline habitat conditions in biodiversity conservation (Suzuki and Parker 2019). Concurrently, against the dual backdrop of global climate change and biodiversity conservation, a growing body of research has called for incorporating carbon stocks into conservation‐planning targets to realize co‐benefits for climate mitigation and species protection. Soto‐Navarro et al. (2020) mapped global “co‐benefits hotspots” for carbon and biodiversity, highlighting the importance of integrating both objectives into conservation policy and spatial planning in selected regions. Moreover, with the rapid expansion of citizen‐science data platforms, much existing research has focused on widely distributed taxa (e.g., birds) for which occurrence data are relatively accessible. By contrast, for rare and endangered animal and plant taxa—where data are difficult to obtain—there have been relatively few studies that assess conservation gaps from the combined perspectives of species distributions, habitat quality, and carbon stocks.

In Hubei Province, existing reserves are clustered in the west, while northeastern and southeastern areas remain under‐protected, leading to an imbalanced spatial distribution (Wang et al. 2010). Furthermore, the current reserve network falls short of the area required to meet the 30% conservation target outlined in the Kunming‐Montreal Framework. Huang et al. (2012) identified the Qinling and adjacent southern regions (including western Hubei) as endemic plant hotspots requiring urgent attention. Some nature reserves in Hubei also suffer from misaligned zoning, as their establishment did not fully account for potential species distributions or human disturbance. Tang et al. (2021) addressed this at a local scale by modeling the distributions of seven mammal species and applying Marxan to optimize Houhe National Nature Reserve. Despite increasing attention to biodiversity conservation, few studies have carried out systematic conservation planning across the entire Hubei Province that target rare and endangered plants from multiple perspectives—namely species distributions, habitat quality, and carbon storage—under both current and future scenarios. This study, based on the SSP1‐2.6 climate scenario, employed an ensemble modeling approach to predict the potential suitable habitats of 10 protected plant species in Hubei Province for the years 2020, 2030, and 2060. In addition, we simulated province‐wide habitat quality and carbon storage to reveal the relationships among protected species' potential distributions, habitat quality, and carbon storage, and to identify conservation‐gap areas from a multi‐perspective viewpoint. Specifically, we aim to answer the following key questions: (1) How will habitat quality and carbon storage change within the study area? (2) What are the current and future geographic distribution patterns of the 10 protected plant species in the study area? (3) How will fragmentation trends of species' potential distributions and habitat quality evolve? (4) From the combined perspectives of species, habitat, and carbon storage, where are the conservation‐gap areas within the study region?

## 2 Materials and Methods

### 2.1 Study Area

**FIGURE 2:**
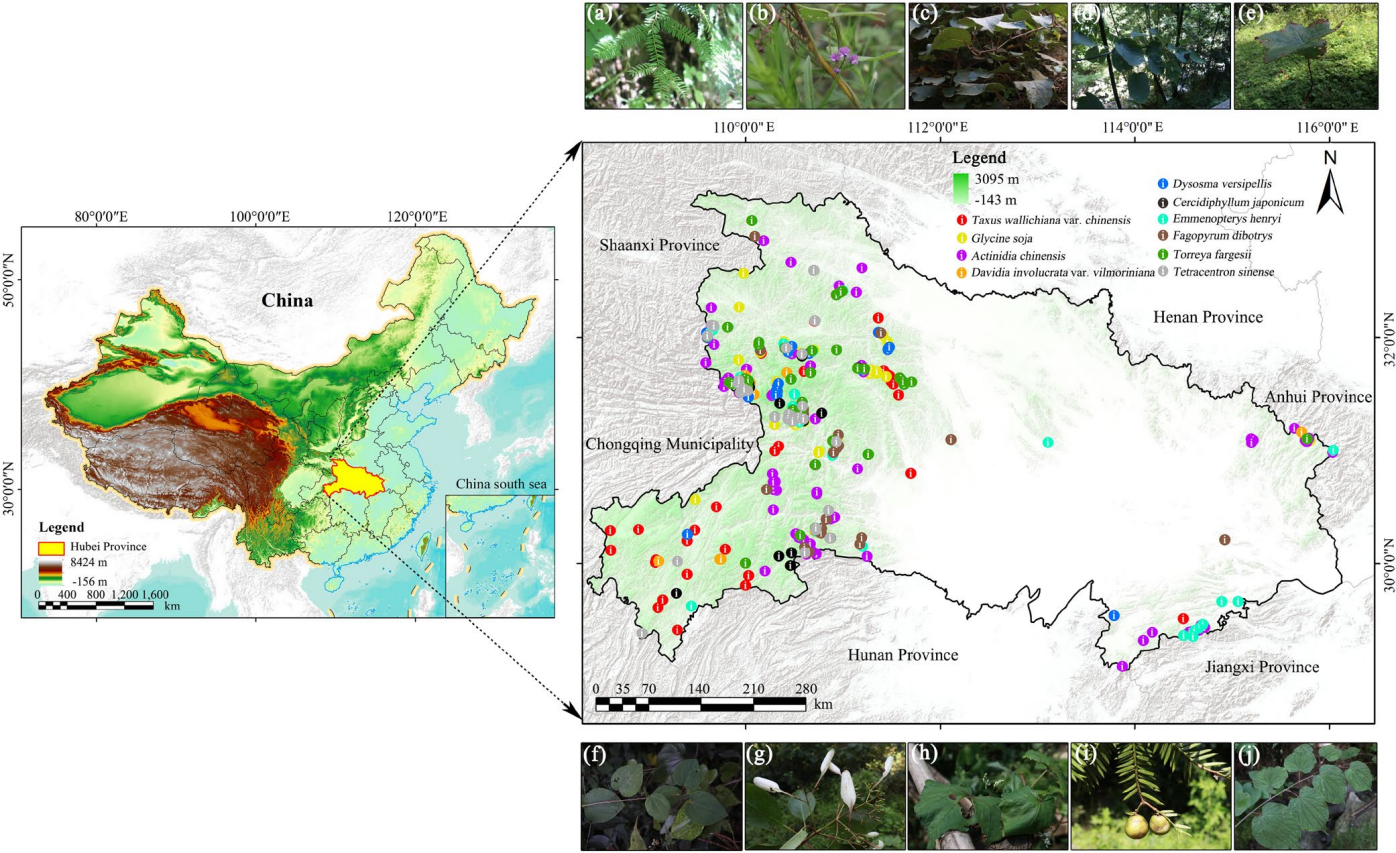
Study area location (Revision No. GS (2024) 0650) (a. *Taxus wallichiana* var. *chinensis*, b. *Glycine soja* , c. *Actinidia chinensis* , d. *Davidia involucrata* var. *vilmoriniana*, e. *Dysosma versipellis*, f. *Cercidiphyllum japonicum* , g. *Emmenopterys henryi*, h. *Fagopyrum dibotrys*, i. *Torreya fargesii*, j. *Tetracentron sinense*).

Hubei Province (29°01′–33°06′ N, 108°21′–116°07′ E) is located in central China and belongs to the subtropical monsoon climate zone, covering a total area of approximately 185,900 km^2^ (DNRHP 2025). Bordered by the Qinling Mountains in the west and the Tongbai–Dabie Mountain ranges in the north, the Yangtze River flows west to east across the province, forming the vast Jianghan Plain through alluvial processes. Hubei lies at the transitional zone between northern and southern China, with a terrain that generally slopes from higher elevations in the west to lower elevations in the east (Figure 2). The province is rich in forest resources with diverse vegetation types. The total area of forested land reaches 92,925 km^2^, accounting for approximately 50% of Hubei's land area (DNRHP 2025). In total, the province hosts 6292 species of vascular plants belonging to 1571 genera and 292 families, among which 154 are listed as nationally protected wild plant species (HPFB 2025). These extensive forest ecosystems—especially in mountainous regions such as western and northeastern Hubei—provide crucial habitats for a wide range of rare and endangered plant species. Owing to its diverse topography and favorable ecological conditions, Hubei serves as a key biodiversity hotspot in south‐central China, offering important refuges for rare and endangered flora (Huang et al. 2012). However, with accelerating socioeconomic development and the ongoing impacts of climate change, the survival of these species is increasingly threatened. Therefore, accurately and effectively identifying protection gaps for these high‐conservation‐value species has become a pressing conservation challenge in the region.

### 2.2 Data Sources and Processing

#### 2.2.1 Species Selection Criteria

To efficiently identify conservation gaps for rare and endangered plants in Hubei Province under limited resources, we prioritized 10 plant species that combine high conservation significance with ecological representativeness. Selection criteria were as follows: (1) National or international conservation status. All selected taxa are listed in national key protection lists or in international conservation registers such as the IUCN Red List and the appendices of CITES, conferring them clear legal or policy priority for protection. For example, *Taxus wallichiana* var. *chinensis* is classified as a first‐class nationally protected species in China and is also listed by IUCN and CITES (Ye et al. 2023). (2) Threat level or conservation value. The chosen species generally possess high conservation value or are currently threatened. *Davidia involucrata* var. *vilmoriniana*, for instance, is considered highly sensitive to future climatic shifts and is projected to experience substantial range contraction. Wild relatives of economically important crops—e.g., *Actinidia chinensis* and *Glycine soja* —represent valuable genetic resources; accurate prediction and protection of their highly suitable wild habitats are therefore important for preserving genetic diversity (Long et al. 2021). (3) Data reliability and model robustness. For each species, we assembled occurrence records from public databases and field surveys. Records underwent coordinate correction, deduplication, and spatial thinning, retaining a statistically meaningful number of independent occurrences (commonly ≥ 30) for species distribution modeling (SDMs). These preprocessing steps reduce spatial bias and strengthen model robustness (Van Proosdij et al. 2016). (4) Ecological and geographic complementarity. Species were deliberately selected to ensure ecological and geographic complementarity across elevation, terrain, climatic and vegetation gradients. The stacked hotspots of rare and endangered plant richness therefore more comprehensively represent Hubei Province's environmental gradients and ecosystem types. This strategy aims to produce a representative spatial surrogate for the protected‐plant assemblage, akin to an “umbrella–connectivity” approach (Dutta et al. 2023).

Ultimately, the 10 focal species selected for this study are: *Taxus wallichiana* var. *chinensis*, *Glycine soja* , *Actinidia chinensis* , *Davidia involucrata* var. *vilmoriniana*, *Dysosma versipellis*, *Cercidiphyllum japonicum* , *Emmenopterys henryi*, *Fagopyrum dibotrys*, *Torreya fargesii*, and *Tetracentron sinense*. These species were chosen to balance conservation importance, modeling feasibility, and ecological coverage, thereby creating a representative and operational framework for identifying and protecting rare and endangered plants in the province.

#### 2.2.2 Species Data

This study selected 10 protected plant species in Hubei Province as target species for potential distribution modeling using SDMs (Thuiller et al. 2009) (Table S1). Species occurrence data were obtained from two primary sources. The first was a series of field biodiversity surveys conducted in mountainous regions and other habitats across Hubei Province from 2010 to 2024. The second source included curated records retrieved from global and national biodiversity databases, specifically the Global Biodiversity Information Facility (GBIF) (https://www.gbif.org/) and the Chinese Virtual Herbarium (CVH) (https://www.cvh.ac.cn/), used to supplement field observations.

Field investigations conducted from 2010 to 2024 combined transect and quadrat sampling methods. Each transect was at least 3 km in length, along which vegetation types and vascular plant species were recorded and photographed. Quadrats were established following the standard phytosociological methods recommended by the International Association for Vegetation Science (Fujiwara 1987), with plot sizes of 20 m × 20 m for trees, 5 m × 5 m for shrubs, and 1 m × 1 m for herbs. Within each quadrat, vegetation was surveyed in three vertical strata: arboreal (≥ 5 m woody plants), shrub (< 5 m woody plants), and herbaceous layers. For each layer, species composition, total cover, density, and abundance were recorded. The “Two Steps” mobile application was used to collect real‐time geographic coordinates (latitude and longitude) of each plot, which were then used as species occurrence data for field surveys. After data integration, a total of 423 occurrence records were compiled, covering 10 species from 10 genera and 9 families (Table S1).

Additional species records were collected from GBIF and CVH databases, with strict filtering criteria: only records from 2010 to 2024 containing essential metadata (species name, image, and geographic coordinates) were retained. This process resulted in an additional 193 valid distribution points for the 10 target species (Table S1).

To ensure spatial accuracy and reduce bias caused by spatial autocorrelation, a data cleaning process was performed on all 616 occurrence records obtained from both sources. Duplicate and geolocation errors were removed. We then conducted spatial autocorrelation testing using the GeoPandas and random libraries in Python. A 1 km buffer was applied to each point, and randomly selected records within spatial proximity were removed to reduce redundancy. The final cleaned dataset consisted of 435 distribution points, which were projected using the Albers Conic Equal Area coordinate system.

The final dataset, including species name, longitude, and latitude, was compiled into an Excel spreadsheet and exported in CSV format for use in the subsequent SDMs modeling workflow.

#### 2.2.3 Environmental Variables

Based on previous studies and the dominant ecosystem types and ecological requirements of protected plant species in Hubei Province, we initially selected 54 environmental variables across four categories: climate, habitat, land cover/land use, and anthropogenic disturbance (Table S2). Climate data included 19 current and future bioclimatic variables, with future climate data obtained from the BCC‐CSM2‐MR model under the CMIP6 framework. All data were sourced from WorldClim version 2.1 (http://www.worldclim.org/). Under the SSP1‐2.6 scenario, the potential distributions of species were predicted for 2030 and 2060. Soil information was derived from the HWSD v2.0 dataset provided by the Food and Agriculture Organization's Global Agro‐Ecological Zones platform (https://gaez.fao.org/). Previous studies indicate that, for most plant species, the 0–30 cm soil layer concentrates root activity and is the critical horizon for water and nutrient uptake (Müllers et al. 2022). Accordingly, we selected eight soil variables from the HWSD v2.0 dataset that are closely associated with root growth in the surface soil (0–30 cm), including soil pH, soil organic carbon (SOC), and soil texture components (clay, sand, and silt), among others. These variables directly affect soil fertility, water‐holding capacity, and nutrient status and therefore represent important environmental determinants of species distributions (Dubuis et al. 2013). Topographic variables—including elevation, slope, and aspect—were derived from the STRM dataset available via Earthdata Search (https://search.earthdata.nasa.gov/) and calculated using ArcGIS 10.2. Habitat greenness was assessed using kernel‐based Normalized Difference Vegetation Index (kNDVI), derived from MODIS datasets (https://modis.gsfc.nasa.gov/). Unlike traditional NDVI, kNDVI incorporates a kernel function to better account for spectral nonlinearities, thereby improving accuracy in complex environments (Camps‐Valls et al. 2021). Land cover and land‐use data were retrieved from the Resource and Environmental Science and Data Center of the Chinese Academy of Sciences (https://www.resdc.cn/). To better align with our study objectives, the original 23 secondary land cover categories were reclassified into six broader types: cropland, woodland, grassland, water bodies, construction land, and unused land (Zhang et al. 2023). Additionally, landscape pattern indices were constructed using Fragstats 4.3 to quantify the impact of habitat patch effects (EHPs) on species distribution (Riva et al. 2024); details are provided in Section 2.4. Anthropogenic disturbance was represented by three gridded datasets: population density, gross domestic product (GDP), and road‐network density. Population density and GDP data were obtained from Wang et al. (2022) and Murakami et al. (2021), respectively, and supplemented by other published sources. Road‐network density was derived from the transportation dataset of the National Catalog Service for Geographic Information (https://www.webmap.cn/). Road features were classified by hierarchy (e.g., primary, secondary, tertiary) and processed in ArcGIS 10.2 using kernel‐density estimation to generate the final road‐density raster. All environmental predictors were subjected to correlation analysis to reduce model overfitting and multicollinearity (Li, Shao, and Jiang 2022). This study used the SDMtune package in R to evaluate the contribution of each environmental variable through repeated modeling. Combined with correlation analysis, variables that were highly correlated were filtered to retain those with greater ecological significance or higher model contribution, thereby effectively avoiding multicollinearity issues (Vignali et al. 2020). The final set of environmental predictors retained for each species is listed in Table S3. Because soil and topographic variables are relatively stable over the temporal horizons considered here, these predictors were held constant in future projections; projected changes were therefore driven by climate variables and land‐use dynamics. To ensure consistency and scientific rigor in spatial analyses, the administrative boundary of Hubei Province was used as the spatial mask. All spatial datasets were projected using the Albers Conic Equal Area coordinate system, with a unified resolution of 1000 m × 1000 m, and were resampled to ensure alignment across raster rows and columns.

### 2.3 Ecosystem Quality Assessment

#### 2.3.1 Future Land Use Simulation

In this study, the Markov‐PLUS model was employed to simulate land‐use changes in Hubei Province for the years 2030 and 2060, based on land‐use data from 2015 and 2020. The simulation incorporated a set of driving factors categorized into three groups: physical geography, anthropogenic disturbance, and locational attributes (Table S4). Model parameters were calibrated according to policy frameworks for future ecological–production–living space, as well as socioeconomic development potential in Hubei Province (Liang et al. 2021).

#### 2.3.2 Habitat Quality Evaluation

To assess habitat quality across Hubei Province, we employed the Habitat Quality module of the InVEST model to calculate a Habitat Quality Index (HQI). The HQI integrates habitat suitability and exposure to threats, with values approaching 1 indicating higher habitat quality. On the basis of land‐use/land‐cover types, threat sources, and habitat‐specific sensitivity to those threats, we constructed province‐wide HQI maps for the years 2020, 2030, and 2060 (Sallustio et al. 2017). The overall habitat quality score for each spatial unit was calculated using the following formula:

(1) $$Q_{\mathrm{xj}}=H_{j}\left[1-\left(\frac{D_{\mathrm{xj}}^{z}}{D_{\mathrm{xj}}^{z}+k^{z}}\right)\right]$$

In this study, the InVEST Habitat Quality module was applied to evaluate the spatial and temporal dynamics of habitat quality in Hubei Province for the years 2020, 2030, and 2060. The model calculates habitat quality *Q*_*xj*_ for each grid cell *x* under land‐use type *j*, which is jointly determined by habitat suitability *H*_*j*_ and the level of habitat degradation *D*_*xj*_. Here, *k* is the half‐saturation constant, typically set to half of the maximum value of *D*_*xj*_, and *z* is a scaling constant, generally fixed at 2.5 (see Appendix S1 for the complete formula and definitions).

Based on land‐use data from 2020, 2030, and 2060, six major threat factors were selected: cropland, construction land, railways, expressways, first‐class highways, and secondary highways. The threat parameters and the sensitivity of different land‐use types to each threat were determined with reference to actual conditions in Hubei Province and related studies (Tian et al. 2025), and are shown in Tables S5 and S6. The habitat quality results were classified into five levels using the equal interval method: high (0.8–1), moderately high (0.6–0.8), medium (0.4–0.6), moderately low (0.2–0.4), and low (0–0.2). Grid cells in the high and moderately high categories were designated as high‐value habitat areas.

#### 2.3.3 Carbon Storage Evaluation

Carbon stock refers to the amount of carbon stored within terrestrial ecosystems; this metric is closely related to an ecosystem's capacity to respond to climate change. In this study, we used the Carbon module of the InVEST model to combine multi‐year land‐use data for Hubei Province for 2020, 2030, and 2060 with four ecosystem carbon pools (aboveground biomass, belowground biomass, soil carbon, and dead organic matter). We calculated mean carbon densities for each land‐cover class to assess the spatial distribution of carbon stocks and their change characteristics. The module computes total carbon stock for the study area by multiplying the carbon density of each land‐use type by its corresponding area and summing carbon across all classes. The specific calculation formula is as follows:

(2) $$c_{i}=c_{i-\text{above}}+c_{i-\text{below}}+c_{i-\text{soil}}+c_{i-\text{dead}}$$

(3) $$C=\sum_{i}^{n}C_{i}\times R_{i}$$

In the formula, *C*_*i*_ represents the carbon density of land‐use type *i*; *C*_*i‐above*_ denotes the aboveground biomass carbon density of land‐use type *i*; *C*_*i‐below*_ refers to the belowground biomass carbon density of land‐use type *i*; *C*_*i‐soil*_ indicates the soil carbon density of land‐use type *i*; and *C*_*i‐dead*_ represents the dead organic matter carbon density of land‐use type *i*. *C* denotes the total carbon stock of the study area, *n* is the total number of land‐use types, and *R*_*i*_ is the area of land‐use type *i*. In the InVEST model, carbon density is one of the key parameters for evaluating carbon storage services and plays a crucial role in the accurate estimation of carbon stocks. The carbon density data used in this study were obtained from published literature (Zhang et al. 2012, 2017; Chuai et al. 2013; Ke and Tang 2019) and were further calibrated according to the actual conditions of Hubei Province (Table S7). Additionally, to facilitate the analysis of the spatial differentiation characteristics of carbon storage, the simulated multi‐period carbon stock results were classified using the Natural Breaks method. The study area was divided into six categories: high carbon storage zone, relatively high carbon storage zone, moderate carbon storage zone, relatively low carbon storage zone, low carbon storage zone, and no carbon storage zone.

### 2.4 Potential Habitat Projections

#### 2.4.1 Developing Multiple Metrics for the Effects of Habitat Patches

Given the increasing spatial resolution of environmental predictor data in SDMs, the ecological effects of within‐grid and surrounding habitat patch characteristics—referred to as EHPs—have received growing attention (Riva et al. 2024). In this study, we used Fragstats 4.3 to calculate multiple patch‐level and landscape‐level metrics related to EHPs based on land‐use data for the years 2020, 2030, and 2060. These indices (Table S8), which capture aspects such as patch area, configuration, and landscape diversity, were incorporated into the modeling process to evaluate their current and future effects on the potential distributions of 10 protected plant species.

#### 2.4.2 Model Development

Species distribution modeling was performed using the “Biomod2” package in R, constructing eight single‐model algorithms: GLM, GBM, CTA, SRE, FDA, ANN, RF, and MaxEnt. For MaxEnt, the “kuenm” package was used to optimize two key parameters—Regularization Multiplier (RM) and Feature Combination (FC). RM values were tested in the range of 0 to 4 with a step size of 0.1 (yielding 40 values), while FC options included 31 different combinations such as L, LQ, LQH, H, LQHP, and LQHPT, resulting in 1240 parameter combinations in total. Model performance was evaluated using Delta Akaike Information Criterion corrected (ΔAICc) and Omission Rate (OR), and the optimal parameter set was selected when ΔAICc < 2 and OR < 5% (Cobos et al. 2019). Previous studies have shown that the choice of feature classes in MaxEnt should be adapted to the sample size of species occurrence points (Elith et al. 2011; Phillips et al. 2006; Phillips and Dudík 2008).

All models used 75% of the occurrence points for training and 25% for validation, with 500 randomly generated background pseudo‐absence points. Each model was run 10 times. Model performance was assessed using TSS, Area Under the Curve (AUC), and Kappa coefficient (Huang et al. 2022; Li, Deng, et al. 2024; Li, Wang, et al. 2024). An ensemble model was constructed using a weighted average method, where TSS > 0.8 was used as the inclusion threshold and TSS values were used to proportionally assign weights—higher TSS implying greater model reliability (Dai et al. 2023). The ensemble probability prediction *P*_EMwmean_ was calculated using the weighted average formula:

(4) $$P_{\text{EMwmean}}=\sum_{i=1}^{N}\omega_{i}\times P_{i}$$

In this equation, *P*_EMwmean_ represents the final predicted probability of the ensemble model; *P*_*i*_ denotes the predicted probability of the *i*‐th individual model; *ω*_*i*_ is the weight assigned to the *i*‐th individual model; and *N* signifies the total number of individual models. Additionally, the constraint $\sum_{i=1}^{N}\omega_{i}=1$ is imposed to ensure the weights sum to unity (for detailed derivation and formulas, see Appendix S1).

To determine the binary habitat suitability of each raster cell for each modeling period, we applied the threshold that maximized the True Skill Statistic (TSS). Raster cells with predicted probabilities above this threshold were assigned a value of 1 (suitable), and those below were assigned 0 (unsuitable). The raster cells classified as suitable (i.e., value = 1) were further categorized into four habitat suitability levels—unsuitable, low, medium, and high—using the natural breaks (Jenks) classification method (Gao et al. 2024). Finally, the areas of high suitability across all 10 protected plant species were overlaid to generate a richness pattern of core potential habitats.

### 2.5 Spatial Patterns of Species Distribution and Habitat Fragmentation

To quantify the multi‐dimensional characteristics of habitat fragmentation under current and future conditions, this study constructed a Composite Landscape Fragmentation Index (CLFI) to assess the impacts of climate and land‐use change on biodiversity (Xu et al. 2023). Four key landscape metrics were integrated: number of patches (NP), patch density (PD), mean patch area (AREA_MN), and aggregation index (AI). Each metric reflects a different dimension of habitat spatial structure and fragmentation intensity. An equal‐weight method (with each index assigned a weight *w* = 1/4) was applied to balance the contribution of individual indicators. The CLFI was calculated using the following formula:

(5) $$\text{CLIF}=\frac{{\mathrm{NP}}_{\mathrm{norm}}+{\mathrm{PD}}_{\mathrm{norm}}+\text{AREA}\_{\mathrm{MN}}_{\text{adjusted}}+{\mathrm{AI}}_{\text{adjusted}}}{4}$$

The index ranges from 0 to 1, with higher values indicating a greater degree of landscape fragmentation (for the full formula, see Appendix S1: Detailed Calculation of the Composite Landscape Fragmentation Index).

### 2.6 Analysis of System Protection Planning From Multiple Perspectives

#### 2.6.1 Assessment of Conservation Gaps Based on Multi‐Perspective Data Integration

The potential highly suitable habitat areas of the 10 plant species simulated by the SDMs under current and future scenarios were overlaid with the high‐value habitat areas and high‐carbon storage zones evaluated by the InVEST model (Xu and Li 2025). Based on this overlay analysis, the study divided the region into eight categories: hotspots of protected plant diversity, species–habitat priority zones, species–carbon sink synergy zones, habitat–carbon sink synergy zones, highly suitable areas, high‐value habitat spaces, high‐carbon storage zones, and non‐significant areas. By integrating the species protection perspective (SPP), habitat protection perspective (HPP), and carbon storage protection perspective (CPP), the study quantitatively identified the conservation gap areas for 10 plant species outside the existing nature reserves in Hubei Province.

#### 2.6.2 Gap Analysis

In this study, the Marxan software, based on the principles of systematic conservation planning (SCP), was employed to identify the optimal set of planning units that meet conservation targets with the minimum number of units and the lowest total cost. This was achieved through iterative calculations and repeated selection using a simulated annealing algorithm (Watts et al. 2009). The mathematical formulation is as follows:

(6) $$\text{Target function}=\sum_{\mathrm{PUs}}\text{Cost}+\mathrm{BLM}\sum_{\mathrm{PUs}}\text{Boundary}+\text{Penalty}\sum_{\text{ConValue}}\mathrm{SPF}$$

In this equation, *Cost* represents the cost factor. In this study, we used the entropy weight method to assign values to GDP and land‐use data to calculate a Human Disturbance Index (HDI), which served as a proxy for this factor. *BLM* refers to the Boundary Length Modifier; *Boundary* indicates the length of the boundary of each planning unit; *SPF* is the Species Penalty Factor; and Penalty denotes the penalty value imposed when conservation targets are not achieved.

The penalty values and Species Penalty Factors (SPF) were assigned based on the protection levels defined by the IUCN, NKPWP, and CITES, as well as the relative importance of habitat quality and carbon storage (Table S9). We optimized the BLM value through sensitivity analysis and selected the best‐performing value to run the Marxan model 100 times. Planning units with a selection frequency (ssoln) greater than 65% and located outside existing nature reserves were identified as conservation gap areas. The conservation gaps identified for the years 2020, 2030, and 2060 were then overlaid, and those units consistently selected as gaps in all 3 years were designated as proposed future conservation areas.

In addition, the calculation of the HDI as a representation of the Cost factor using the entropy weight method is given by the following equation:

(7) $$\mathrm{HDI}=\sum_{j=1}^{n}{\mathrm{HDI}}_{j}\times W_{j}$$

In this equation, *HDI*_*j*_ represents the *j*‐th type of human disturbance factor, which includes GDP and land‐use data. *W*_*j*_ denotes the weight of the *j*‐th human disturbance factor, calculated using the entropy weight method (Cunha‐Zeri et al. 2022). The detailed formula and calculation process are provided in the Appendix S1.

## 3 Results

### 3.1 InVEST Evaluation Results

#### 3.1.1 Habitat Quality Evaluation Results

**FIGURE 3:**
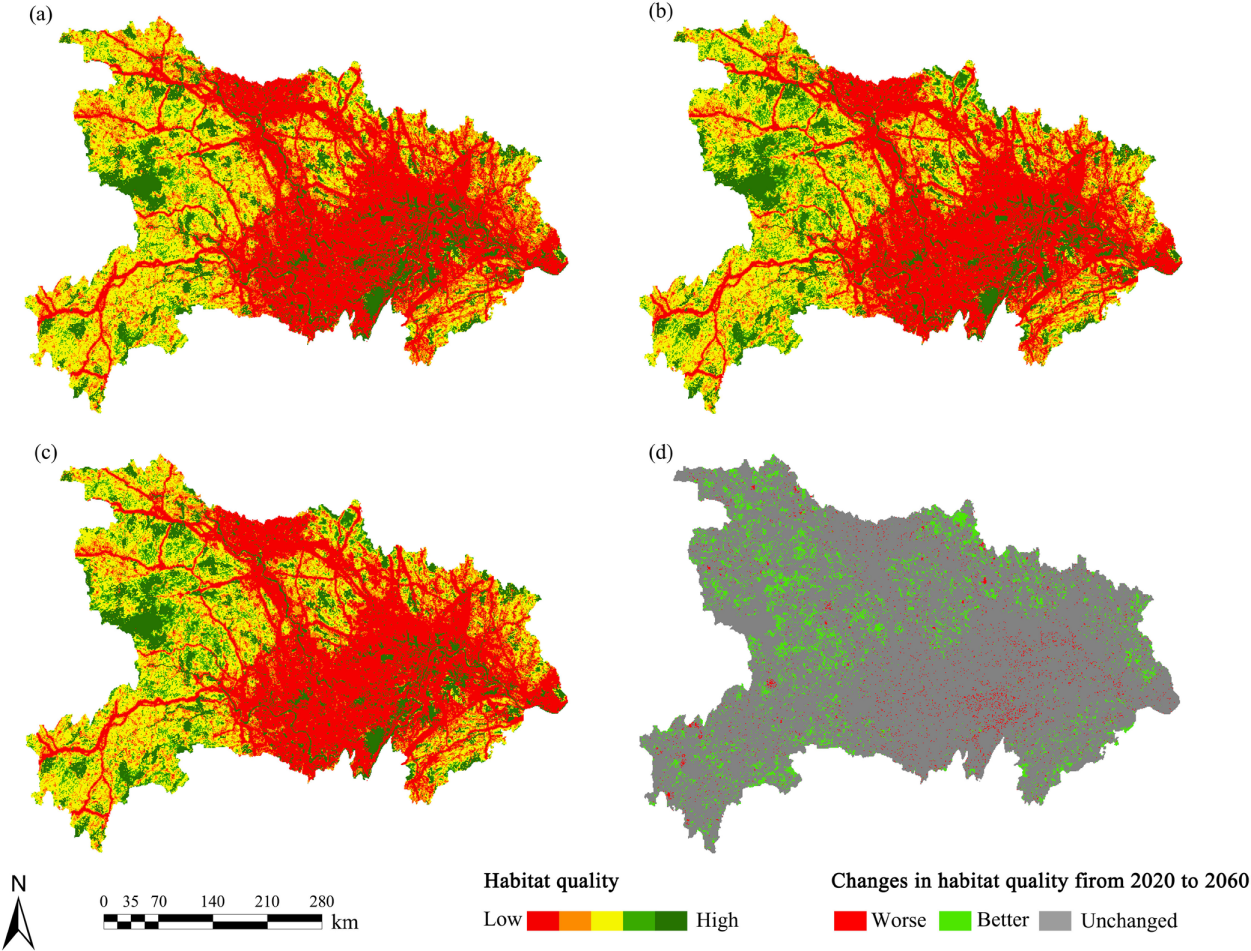
Spatial pattern of habitat quality in Hubei Province (a. 2020, b. 2030, c. 2060, d. Changes in habitat quality from 2020 to 2060).

Between 2020 and 2060, habitat quality in Hubei Province showed a slight upward trend. The average habitat quality index increased from 0.355 to 0.366; however, it remained at a medium to relatively low level overall (Figure 3a–c). At present, approximately 17% of the region is classified as high‐value habitat, while low‐value habitats account for over 56% of the total area. Low‐value habitats are primarily distributed in the central and eastern plains, particularly in urban agglomerations along the Yangtze and Han Rivers, as well as in scattered rural areas nearby. In contrast, mountainous regions in the west and southwest, along with natural rivers and lakes on the plains, exhibit relatively higher habitat quality.

**FIGURE 4:**
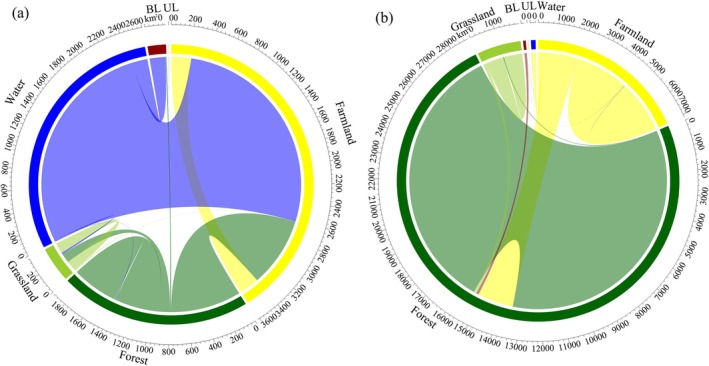
Land‐use change (a. Areas with declining habitat quality from 2020 to 2060, b. Areas with improving habitat quality from 2020 to 2060; UL and BL represent unused land and construction land, respectively).

The overall habitat quality in the region is projected to improve by 2060. Specifically, habitat quality increased in 10.24% of the area, while only 2.33% experienced degradation (Figure 3d). The improved areas are mainly located in transition zones between mountainous and plain regions in the west, north, and south, whereas declines are concentrated around urban agglomerations in the plains and surrounding natural rivers and lakes. In areas with improved habitat quality, the largest land‐use change was in forest land, which expanded by 1948 km^2^. The dominant land conversion type was cropland to forest land, accounting for 1754 km^2^, or 38.70% of the original cropland area within these zones. Notably, 115 km^2^ of construction land was converted to forest land, representing 95.83% of the original construction land area in the improved zones (Figure 4a). In contrast, in areas where habitat quality declined, cropland saw the greatest increase in area, expanding by 3181 km^2^. The primary conversion type was from water bodies to cropland, covering 2405 km^2^, which accounts for 70.71% of the original water body area in the degraded zones. This was followed by the conversion of forest land to cropland, amounting to 774 km^2^, or 55.25% of the original forest area within those regions (Figure 4b).

#### 3.1.2 Carbon Storage Evaluation Results

Based on the InVEST carbon‐stock model, the assessment indicates that the multi‐year mean total carbon stock of Hubei Province is approximately 2.12 × 10^9^ t. By carbon‐stock class, high carbon‐storage zones account for roughly 60% of the province's total carbon stock and are mainly distributed in mountainous and hilly areas with abundant natural vegetation cover; medium carbon‐storage zones account for about 30% and are located in agro‐forest transition areas such as farmland. Between 2020 and 2060, the province's total carbon stock shows a sustained but slow increasing trend, rising from 2.11 × 10^9^ t in 2020 to 2.13 × 10^9^ t in 2060. This change indicates that Hubei's overall carbon‐sink capacity is expected to remain steadily enhanced in the future.

**FIGURE 5:**
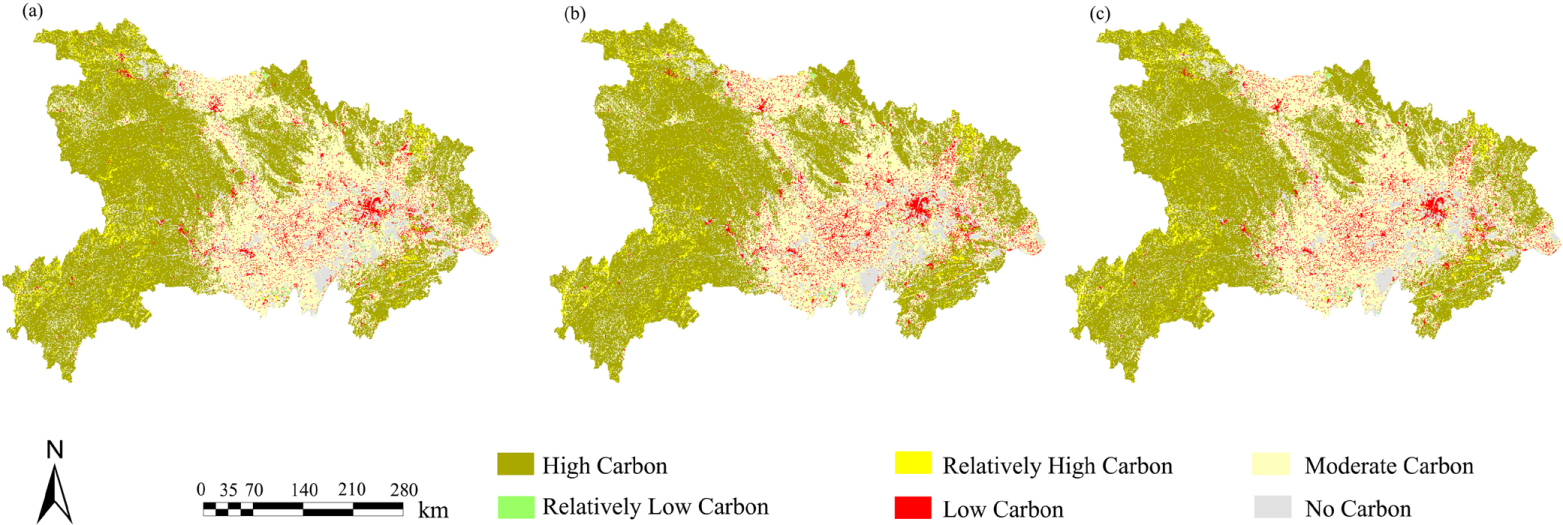
Spatial distribution pattern of carbon stocks in Hubei Province (a. 2020, b. 2030, c. 2060).

In terms of spatial patterns, Hubei's carbon stocks display a “west‐high, central‐medium, east‐low” differentiation (Figure 5a–c). The western, northeastern, and southeastern mountainous regions—characterized by higher elevation, humid climate, and high forest cover—host the province's most concentrated carbon stocks; by contrast, central and central‐eastern areas, which are strongly affected by urban expansion and agricultural activities, are dominated by cropland, built‐up land, and water bodies and therefore generally exhibit moderate to low carbon stocks. From 2020 to 2060, land‐use changes produced spatially heterogeneous changes in carbon stock. The continued expansion of built‐up areas has converted portions of farmland and forest and led to declines in carbon stock in some locations; at the same time, ecological restoration and conversion of cropland to forest have restored and increased forest area in other locations, thereby contributing to an overall rise in carbon stock.

### 3.2 Potential Suitable Habitats for 10 Protected Plant Species Within the Study Area

#### 3.2.1 Ensemble Model Optimization and Evaluation Metrics

The MaxEnt model was optimized using the “kuenm” package, and species‐specific optimal parameters were obtained for all 10 protected plant species (Table S10). The optimized MaxEnt models were then integrated with the other individual models for simulation. Finally, only those individual models with a TSS greater than 0.8 were selected to construct the ensemble model using a probability‐weighted averaging method. The results showed that the average AUC, TSS, and Kappa values across the 10 species were 0.986, 0.927, and 0.640, respectively, while the minimum values were 0.962, 0.853, and 0.412, respectively (Table S11). These results indicate that the models performed well and are suitable for predicting the diversity patterns of potential suitable habitats for protected plant species.

#### 3.2.2 Environmental Variable Importance

**FIGURE 6:**
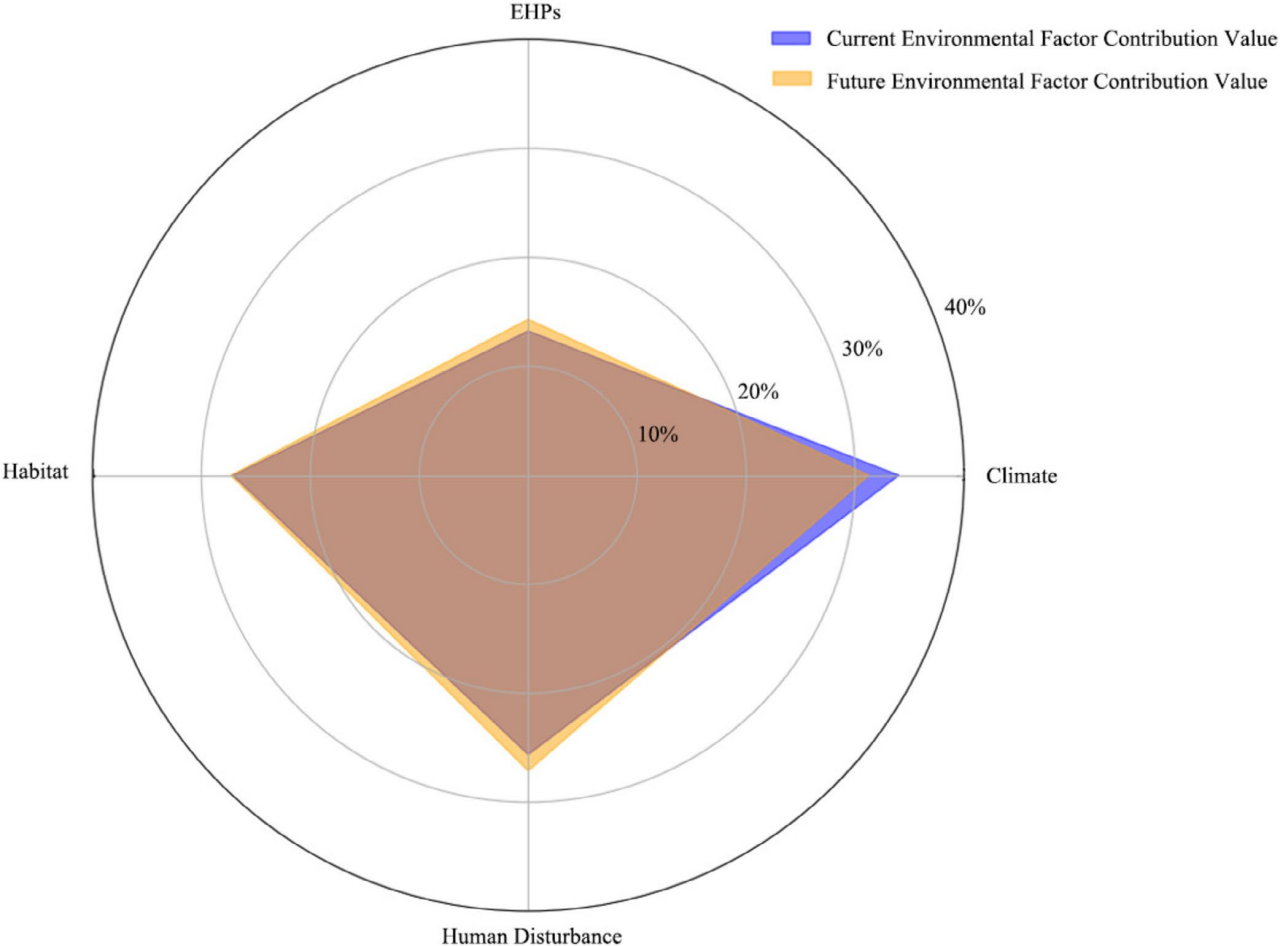
Current and future contribution values of four types of environmental factors: Climate, EHPs, habitat, and human disturbance.

The environmental variables were categorized into four types: climate, EHPs, habitat, and human disturbance. For the 10 protected plant species, the current average contribution of each variable type to the model was ranked as follows: climate (34.03%), habitat (27.11%), human disturbance (25.63%), and EHPs (13.24%). Under future scenarios, the average contribution of anthropogenic disturbance, EHPs, and habitat factors increased by 1.49%, 1.07%, and 0.19%, respectively, while the contribution of climatic factors decreased by 2.76% (Figure 6). Specifically, the distributions of *Taxus wallichiana* var. *chinensis*, *Glycine soja* , *Actinidia chinensis* , *Davidia involucrata* var. *vilmoriniana*, and *Cercidiphyllum japonicum* were primarily driven by climatic variables. In contrast, *Torreya fargesii* and *Tetracentron sinense* were mainly influenced by habitat factors, while the distributions of *Emmenopterys henryi* and *Fagopyrum dibotrys* were mainly influenced by EHPs, and that of *Dysosma versipellis* was relatively sensitive to changes in anthropogenic disturbance. Under future scenarios, the contribution of climate factors to the distributions of *Davidia involucrata* var. *vilmoriniana* and *Torreya fargesii* increased the most, while EHPs showed the greatest increase in influence on *Cercidiphyllum japonicum* , *Fagopyrum dibotrys*, and *Tetracentron sinense*. Additionally, the distributions of *Taxus wallichiana* var. *chinensis*, *Glycine soja* , and *Actinidia chinensis* became increasingly dependent on original natural habitats, whereas *Emmenopterys henryi* and *Dysosma versipellis* exhibited growing sensitivity to human disturbance (Table S12).

#### 3.2.3 Simulated Potential Suitable Habitat Distribution Results

The ensemble model was used to simulate the current and future potential distributions of the 10 protected plant species (Figure S1). Among them, *Fagopyrum dibotrys* (21,065 km^2^), *Torreya fargesii* (21,064 km^2^), and *Actinidia chinensis* (16,847 km^2^) exhibited the largest overall potential distribution areas. In terms of spatial patterns, the potential suitable habitats for most species were mainly concentrated in the western part of Hubei Province, particularly along the Qinling–Daba Mountains, the Wuling Mountains, and their associated ranges. Some species also showed scattered distributions in the northeastern Dabie–Tongbai Mountains and the southeastern Mufu Mountain areas. Temporally, by 2060, the potential distribution areas of five species will have expanded. The expansion percentages were as follows: *Glycine soja* (126.73%), *Cercidiphyllum japonicum* (37.47%), *Taxus wallichiana var. chinensis* (35.99%), *Emmenopterys henryi* (17.00%), and *Davidia involucrata var. vilmoriniana* (7.50%). In contrast, the remaining five species exhibited contractions in their potential habitats: *Torreya fargesii* (−46.96%), *Actinidia chinensis* (−41.11%), *Fagopyrum dibotrys* (−40.50%), *Tetracentron sinense* (−27.61%), and *Dysosma versipellis* (−0.05%) (Table S13).

**FIGURE 7:**
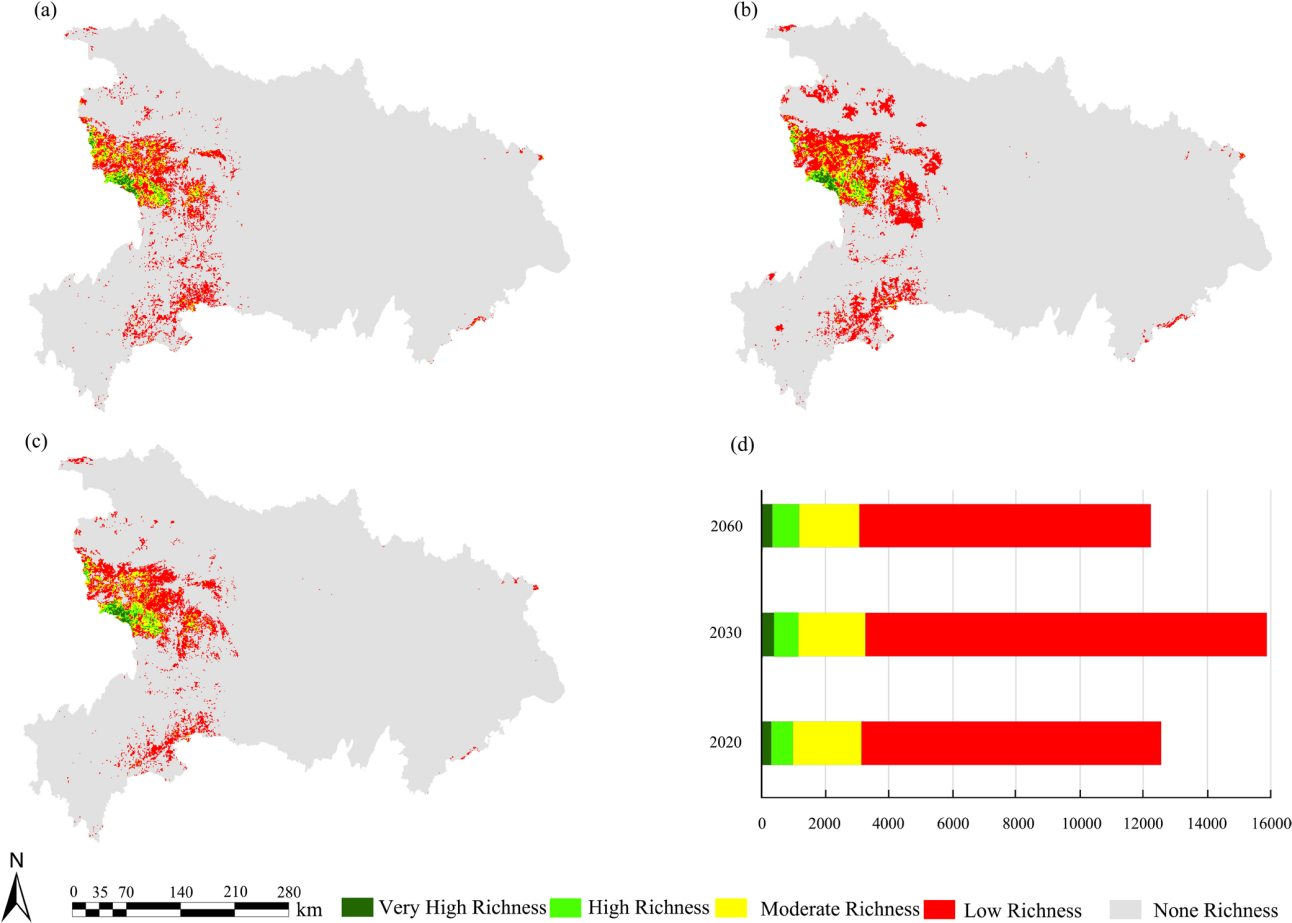
Distribution patterns of richness for 10 protected plant species. (a. 2020, b. 2030, c. 2060, d. Changes in richness area from 2020 to 2060).

By overlaying the high potential suitable habitats of the 10 protected plant species, a richness map of the core potential distribution areas was generated and classified into five categories: high richness (7–10 species), relatively high richness (5–6 species), moderate richness (3–4 species), low richness (1–2 species), and no richness (0 species) (Figure 7a–c). Areas with high species richness were primarily concentrated in the Shennongjia region and its surrounding mountainous areas. However, under future changes in climate and land cover, the total area of high richness zones is projected to decline by 2.6% (Figure 7d).

### 3.3 Analysis Results of Species and Habitat Quality Fragmentation

**FIGURE 8:**
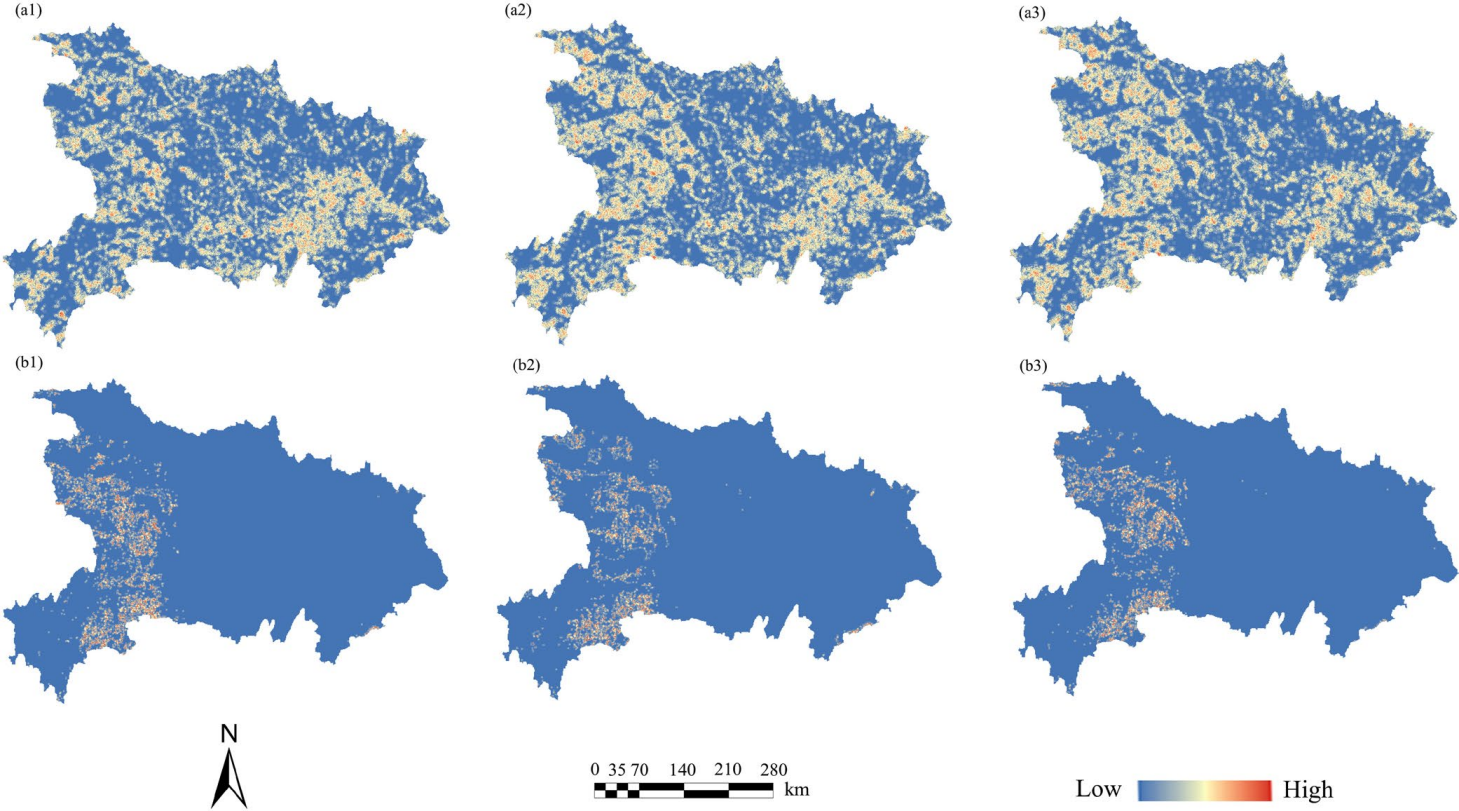
Distribution patterns of CLFI in species' high‐potential distribution areas and high‐value habitat spaces (a. High‐value habitat spaces, b. High‐potential distribution areas of species; 1–3 represent the geographic distribution of CLFI in 2020, 2030, and 2060, respectively).

The CLFI of high potential species distribution areas and high‐quality habitat spaces was assessed from 2020 to 2060. For high potential species distribution areas, the average CLFI decreased from 0.044 in 2020 to 0.037 in 2060, indicating a general decline in fragmentation. Notably, areas with high fragmentation values gradually shifted toward the central and low‐altitude mountainous regions in western and southwestern Hubei (Figure 8b1–3). In contrast, the fragmentation of high‐quality habitat areas exhibited an opposite trend, with the average CLFI slightly increasing from 0.265 in 2020 to 0.277 in 2060. Spatially, fragmentation decreased in the plains and around the Wuhan metropolitan area. However, in mountainous–farmland ecotones that experience strong human disturbance, habitat quality improved over time, but fragmentation simultaneously intensified (Figure 8a1–3).

### 3.4 Multi‐Perspective Analysis of Systematic Conservation Planning

#### 3.4.1 Biodiversity Hotspot Analysis From a Coupled Species–Habitat–Carbon‐Stock Perspective

**FIGURE 9:**
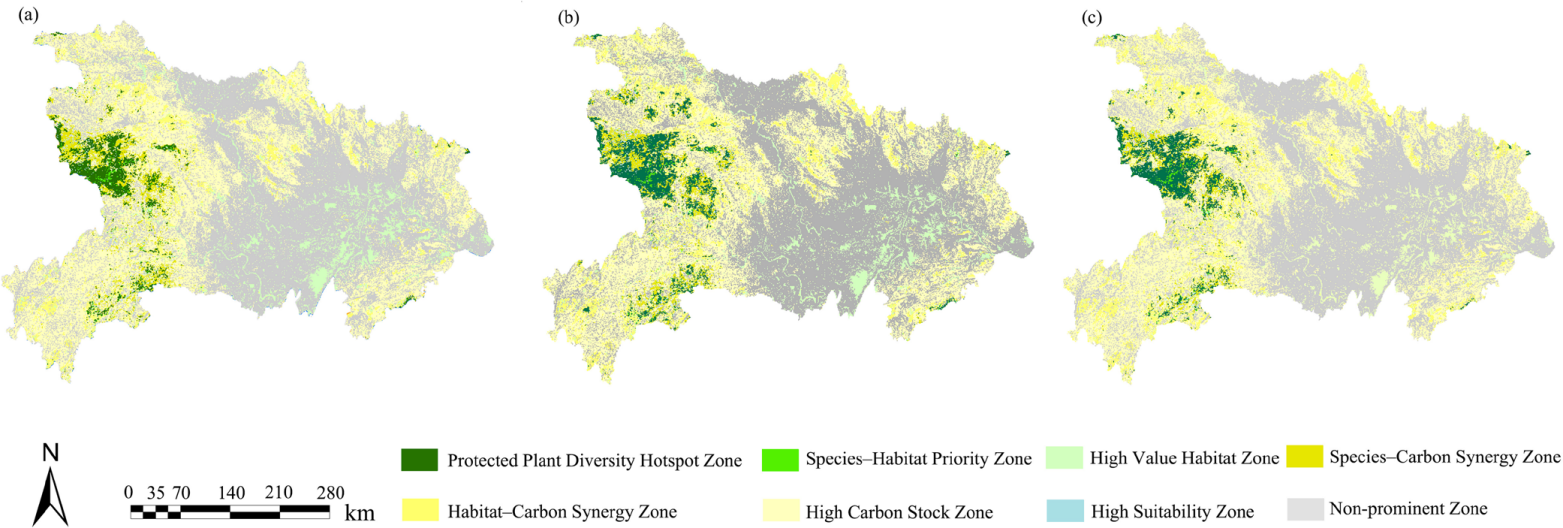
Overlay analysis map based on species–habitat–carbon storage (a. 2020, b. 2030, c. 2060).

In the current study area, 2.94% of the land represents the overlap of protected species' highly suitable potential habitats, high‐value habitat spaces, and high‐carbon storage zones—i.e., hotspots of protected plant biodiversity—most of which are concentrated around Shennongjia National Park. 3.04% of the area is classified as species–carbon synergy zones, 0.28% as species–habitat priority zones, and 6.57% as habitat–carbon synergy zones. Meanwhile, 36.45% of the area corresponds to high‐carbon storage zones only, 7.19% to high‐value habitat spaces only, and 0.48% to highly suitable potential habitat zones only (Figure 9). Over 98% of the protected‐plant biodiversity hotspots are dominated by forest and grassland, two land‐cover types that are relatively little affected by human activities. In the future, the area of protected‐plant biodiversity hotspots is projected to increase by 0.78%, and the land‐use composition of species–habitat priority zones, species–carbon synergy zones, and highly suitable potential habitat zones only shows a degree of congruence with the biodiversity hotspots, with over 96% of these areas consisting of forest and grassland.

#### 3.4.2 Conservation Gap Analysis Through Systematic Conservation Planning

In this study, systematic conservation planning and spatial prioritization were conducted across 28,959 planning units, each delineated by administrative village boundaries, based on different conservation targets. As the conservation targets increased, both conservation cost and boundary length also rose accordingly. Therefore, a BLM of 0.001 was selected as the most appropriate value, balancing cost and boundary length (Figure S2). The SPF was determined based on species conservation status according to IUCN, NKLPS, and CITES classifications, as well as habitat quality levels (Table S9).

**FIGURE 10:**
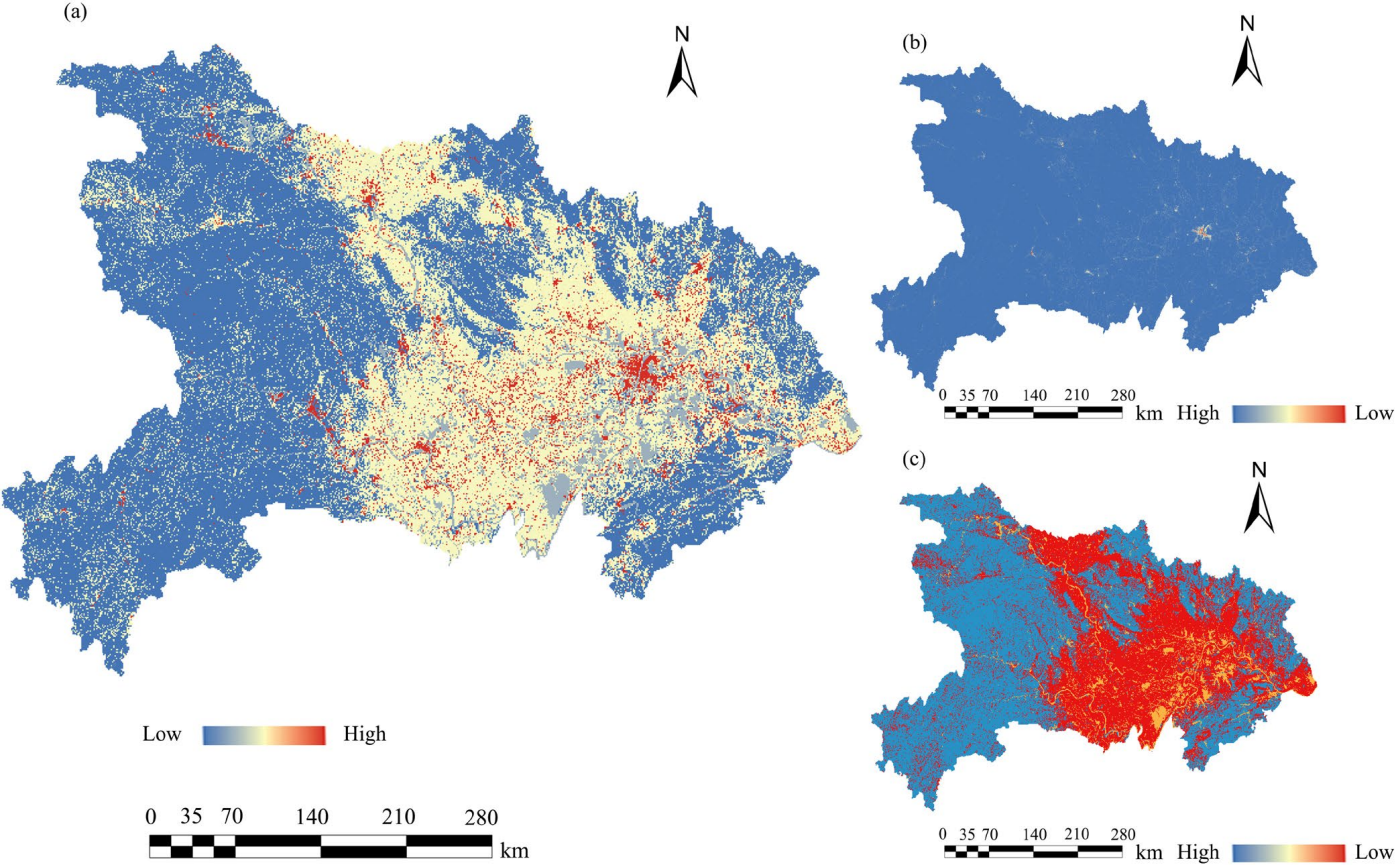
Geographic distribution of HDI in the study area (a. HDI, b. Gridded GDP, c. Land‐Use Types).

The cost value was represented by the HDI, calculated for the years 2020, 2030, and 2060 (Figure 10). The entropy‐weighted distribution of HDI components indicated that in 2020, the entropy weights of GDP and land‐use were 0.681 and 0.319, respectively. These weights were adjusted to 0.699 and 0.301 in 2030, and further evolved to 0.702 and 0.298 in 2060 (Figure S3). This trend suggests a continuous increase in the relative importance of the GDP component, whereas the significance of the land‐use factor shows a decreasing trajectory over time.

**FIGURE 11:**
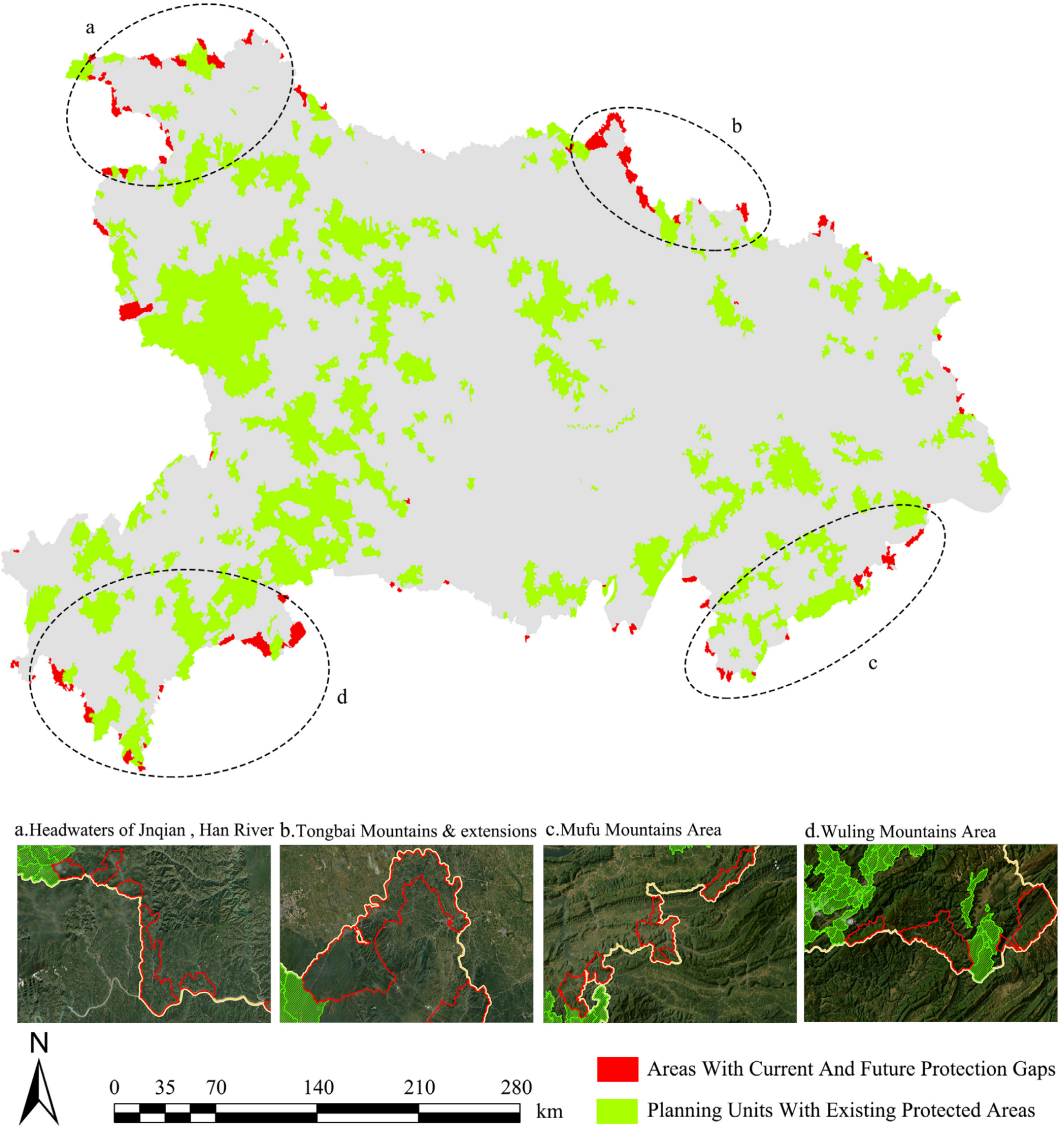
Conservation gap areas identified by multi‐period systematic conservation planning (a. Along the headwaters of the Hanjiang River's tributaries—Jinqian River and Tianhe River, b. Tongbai Mountain and its extensions, c. Along the Mufu Mountain range, d. Along the Wuling Mountain range).

The final results of the systematic conservation planning simulations revealed gaps in the delineation of conservation areas in both current and future scenarios within the study region. Specifically, 2878 planning units are currently designated as part of existing nature reserves, while 277 units remain as conservation gaps (Figure S4a). In 2030 and 2060, an additional 366 and 359 planning units, respectively, were identified as conservation gaps (Figure S4b,c). However, due to projected changes in potential species distributions, variations in habitat quality, and the continuous increase in the HDI, relying solely on results from a single time period may compromise the accuracy of identifying conservation gaps. Therefore, we overlaid the conservation gap results for 2020, 2030, and 2060, and identified a total of 219 planning units that consistently remained unprotected across all three periods. These persistent conservation gaps account for 8% of the currently protected units. Spatially, most of these persistent conservation gaps are located along provincial boundaries and can be broadly categorized into four regions: (1) the mountainous corridor along the tributaries of the Han River, including the Jinqian River and Tianhe headwaters in Shiyan City, bordering Shaanxi Province (Figure 11a); (2) the Tongbai Mountains and surrounding ridges in Suizhou City, bordering Henan Province to the north (Figure 11b); (3) the Mufu Mountain range within Xianning and Huangshi cities to the southeast (Figure 11c); and (4) the Wuling Mountains in Enshi and adjacent hilly areas along the border with Chongqing Municipality to the southwest (Figure 11d).

## 4 Discussion

### 4.1 Future Changes and Regional Differences in the Habitat Quality of Hubei Province

Future habitat quality in Hubei Province is expected to show a gradual improvement, reflecting the initial effectiveness of policies aimed at promoting the coordinated advancement of ecological conservation and economic development (Wei et al. 2025). However, the core challenge of a “low‐level improvement” should not be overlooked. Despite an overall positive trend, much of the land in the study area remains classified as medium or low‐value habitats, with limited improvement anticipated in the future. This underscores the difficulty of regional ecological restoration, particularly in human‐dominated landscapes such as the Jianghan Plain and the urban agglomerations along the Yangtze River, where habitat degradation has become entrenched (Tian et al. 2025). Understanding spatial heterogeneity is essential for interpreting ecological dynamics and informing ecosystem conservation strategies (Zhang et al. 2022). The current spatial distribution of habitat quality indicates that western mountainous areas and natural river–lake systems maintain relatively high habitat quality due to low levels of human disturbance. In contrast, the central and eastern plains, dominated by urban clusters, have become ecological “lowlands” due to intensive development. Notably, this pattern of divergence is projected to intensify in the future. Habitat quality improvements are primarily concentrated in transitional zones of mountainous regions in the west and north, while areas of degradation overlap significantly with existing ecological weak spots, such as urban zones and the peripheries of river and lake systems. This emerging pattern of “the strong get stronger, the weak get weaker” may exacerbate inequalities in the provision of ecological services (Zeng et al. 2023). Land‐use change analysis further explains these effects (Li, Dong, et al. 2022). In areas with rising habitat quality, conversions from cropland to forest (1754 km^2^) and from built‐up land to forest (115 km^2^) suggest the positive effects of China's future “Dual‐Carbon Pathway” strategies, including cropland retirement and urban–rural ecological restoration (Ma et al. 2024). In contrast, areas with declining habitat quality are marked by large‐scale conversions from water bodies to cropland (2405 km^2^) and from forest to cropland (774 km^2^), revealing key concerns. The former suggests that under future farmland protection and food security policies, wetlands in flat, water‐abundant regions such as the Jianghan Plain may be extensively reclaimed for agriculture, potentially undermining the water retention and ecological corridor functions of the Yangtze River Basin (Mao et al. 2018). The latter reflects the pressure of agricultural reclamation on the margins of mountainous areas. These two types of land‐use conversion are mainly concentrated around rivers and cities in plain regions and align closely with areas of habitat degradation, highlighting the ongoing conflict between agricultural development and ecological conservation. In summary, habitat quality in the study area is characterized by localized improvement but overall vulnerability, with spatial heterogeneity becoming increasingly pronounced. To transition from “low‐level improvement” to “high‐quality equilibrium,” efforts must focus on overcoming the ecological challenges in the plains and mitigating the degradation risks associated with anthropogenic land‐use changes.

### 4.2 Spatiotemporal Variation and Regional Patterns of Carbon Storage in Hubei Province

The overall carbon storage in Hubei Province is projected to increase in the future. The spatial distribution of carbon stocks is closely associated with vegetation cover: areas with higher vegetation cover and vigorous plant growth generally have higher carbon stocks, whereas areas with sparse or degraded vegetation show lower carbon stocks (Mo et al. 2023). The observed trend in Hubei's total carbon stock indicates that ecological restoration and forest recovery policies have yielded some effects. However, the magnitude of the increase is limited, and regional carbon‐sequestration potential has not been fully realized (Wang et al. 2021). Spatially, carbon stocks display a west‐high, east‐low pattern. In western mountainous areas, concentrated forest cover and weaker human disturbance result in higher carbon stocks. Central and east‐central plains—affected by urban expansion, encroachment of agricultural land, and local natural conditions—generally exhibit relatively low carbon stocks (Chuai et al. 2022). Future scenario projections indicate that high carbon‐stock areas will be concentrated in ecological restoration zones, while declines are more common in rapidly urbanizing areas (Zhu et al. 2022). Previous studies report that land‐use change is the main driver of carbon‐stock variation. Expansion of built‐up land has occupied some cropland and forest, reducing overall carbon‐sequestration function, whereas cropland‐to‐forest conversion and ecological restoration have increased total carbon stocks (Zhang, Luo, et al. 2024). Overall, Hubei's carbon‐stock dynamics reflect the combined influence of urban expansion and ecological restoration; in the future, optimizing land‐use structure and strengthening forest management are needed to sustain improvements in carbon‐sequestration capacity.

### 4.3 Combined Model Reveals the Evolution of Distribution Patterns for 10 Protected Plant Species Dominated by Climate

**FIGURE 12:**
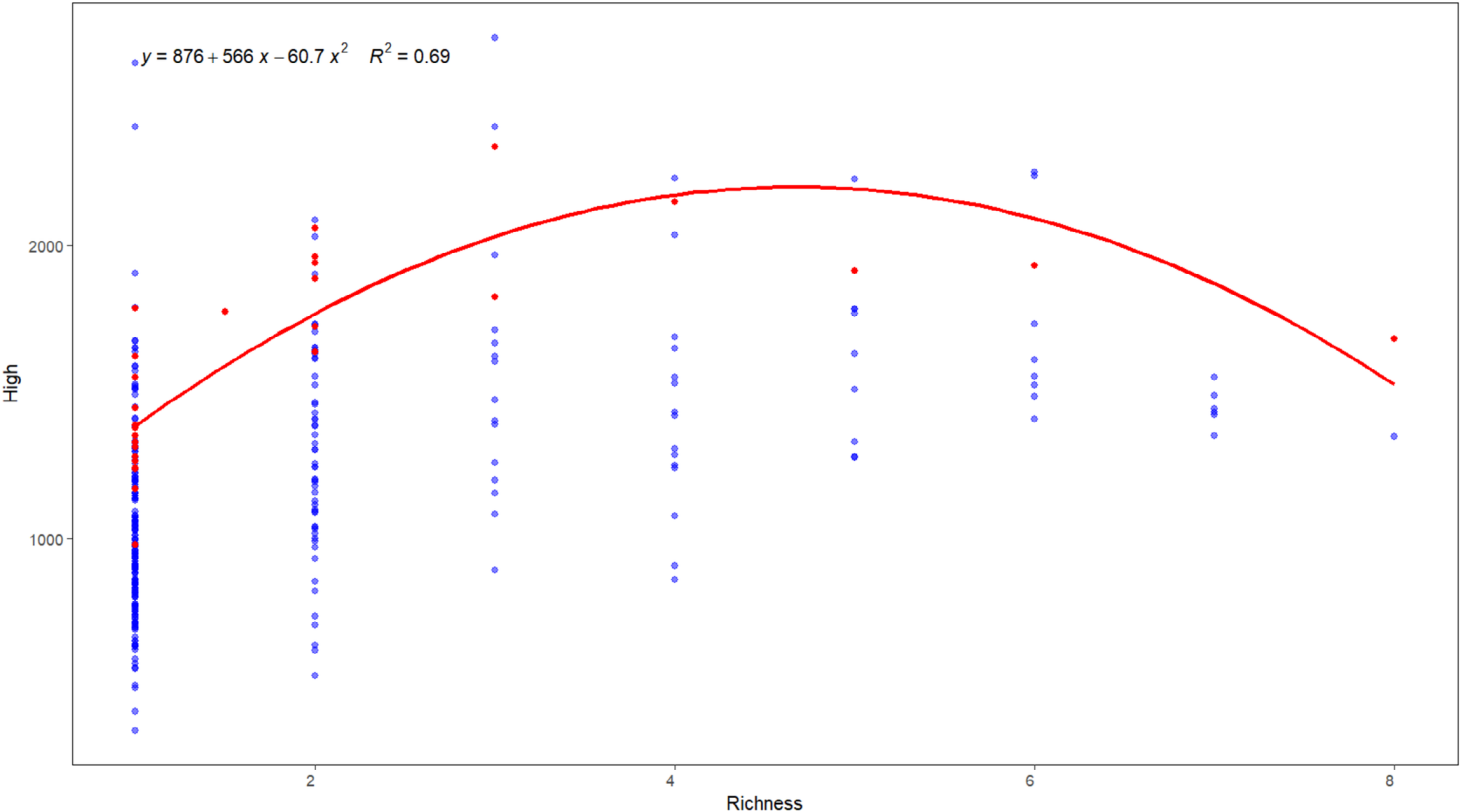
Fitted relationship between elevation and species richness.

Compared with individual models, ensemble models generally exhibit higher overall performance due to their ability to capture complex functions and interactions among covariates (Valavi et al. 2022). In this study, we evaluated the predictive accuracy and overfitting risk of multiple algorithms. The most appropriate models were selected based on these assessments and integrated using a probability‐weighted averaging method. The resulting ensemble model demonstrated lower overfitting risk and high predictive accuracy, as evidenced by strong average values across AUC, TSS, and Kappa metrics. Guo et al. (2020) also found that ensemble models, compared to single models, reduce dependency on sample size, lower the risk of overfitting, and improve the representation of species–environment relationships. Additionally, Yang et al. (2019) conducted a field survey of rare and endangered plant species in eight nature reserves in northwestern Hubei and found that species richness peaked at elevations between 900 and 1700 m. This finding is consistent with the hump‐shaped relationship between species richness and elevation observed in this study, derived from quantile classification of the potential high‐suitability habitats of 10 protected plant species (Figure 12). This again confirms the reliability of the model predictions in reflecting species distribution patterns. The pattern of maximum species diversity at mid‐elevations has been widely documented in plant community studies (Naiman and Décamps 1997). This phenomenon may be attributed to factors such as ecological niche diversity associated with elevation gradients and the dynamic cycling of water resources (Bertuzzo et al. 2016; Rawat et al. 2021).

Analysis of environmental drivers revealed that climatic factors remain the primary determinants of the potential distribution of the 10 protected plant species, both in the current and future scenarios. This finding is consistent with the central role of climate in shaping plant biogeographical patterns at the global scale (Cabra‐Rivas et al. 2016). Notably, Riva et al. (2024) emphasized the importance of incorporating EHPs into SDMs to improve model performance and to better capture environmental influences at finer spatial scales. In this study, we included EHPs as one of the environmental predictors in the SDMs and found that their contribution rate significantly increased in future scenarios, making them the second most important variable group. This suggests that EHPs play a critical role in shaping the potential distribution of protected plant species. In contrast, Li et al. (2025) modeled the potential distribution of *Taxus wallichiana* var. *chinensis* in Hubei Province without considering EHPs, which resulted in a model overly influenced by climate alone, predicting a pronounced contraction of suitable habitat in the southern region. Compared with those results, the integrated use of multidimensional environmental predictors in our study yielded more ecologically plausible outcomes. Furthermore, the growing influence of EHPs may reflect the increasing importance of patch‐ and landscape‐scale characteristics, such as habitat area, configuration, and diversity (Riva et al. 2024).

The simulation results revealed a distinct spatial differentiation in the current potential distribution of the 10 protected plant species, characterized by a “more in the west, less in the east” pattern. Among them, *Fagopyrum dibotrys*, *Torreya fargesii*, and *Actinidia chinensis* had the largest potential habitat areas, with most high‐suitability zones concentrated in the Qinba Mountains in western Hubei. This distribution pattern corroborates the role of the mountainous regions in western Hubei as both Quaternary glacial refugia and monsoonal corridors, which have preserved diverse floristic components and harbored a rich array of rare and endangered plant species (Yang et al. 2019). In particular, the complex topography of the Qinling–Daba mountain range has served as a sanctuary for ancient and relict species—a phenomenon referred to as the “Eastern Sichuan–Western Hubei Endemism Center,” a unique floristic subregion (Zhao, Li, et al. 2024). By 2060, the potential distributions of different plant species show a polarized trend of “expansion versus contraction.” Expanding species such as *Glycine soja* and *Cercidiphyllum japonicum* are projected to shift toward lower elevations and higher latitudes, supporting the hypothesis that climate warming may increase the extent of suitable habitats for certain species (Pecl et al. 2017). In contrast, species such as *Fagopyrum dibotrys* and *Dysosma versipellis* exhibit a marked contraction of their potential ranges, highlighting their sensitivity to anthropogenic disturbances. This aligns with global findings that habitat loss is a key driver of range reductions among threatened herbaceous plants (Bedair et al. 2021). Paradoxically, *Fagopyrum dibotrys*, which is currently the most widely distributed species, faces a substantial risk of range contraction, indicating that widespread species may, because of factors such as niche dependency, not necessarily be resilient to climate change (Pacifici et al. 2015).

### 4.4 Future Species Distribution and Habitat Quality Fragmentation Will Show Increasing Divergence

The significant decrease in the mean CLFI of the species' high potential distribution areas suggests two main implications. On the one hand, it indicates enhanced connectivity among potential future habitats. On the other hand, the loss of mid‐ to low‐elevation mountain habitats—where current core distribution areas are located—has led to an upward shift in the core distribution zones toward higher elevations, thereby reducing overall landscape fragmentation. This trend is consistent with the “elevational range shift theory under climate change,” which posits that climate warming causes fragmentation and loss of low‐elevation habitats, prompting species to migrate upward. As a result, species tend to aggregate at higher altitudes, and the remaining patches resemble isolated “habitat islands” (Parmesan and Yohe 2003; Shafer et al. 2001). In contrast, the CLFI values of high‐quality habitat areas have increased, with intensified fragmentation occurring primarily in the transitional zones between mountainous areas and farmlands in the western part of the study area. Although habitat quality has improved in these regions—partly due to ecological restoration policies such as reforestation on sloped farmland—the improved habitat patches are likely secondary forests regenerated from croplands, which remain noticeably fragmented. Furthermore, the complete restoration of high‐quality forest ecosystems requires time. In addition, edge effects resulting from agricultural activities and urban expansion have further disrupted habitat continuity. These factors together have given rise to a phenomenon where “habitat quality increases but connectivity decreases” in certain areas. Haddad et al. (2015) analyzed global forest cover and revealed that 70% of existing forests, located within 1 km of their edges, are experiencing degradation impacts due to fragmentation.

### 4.5 Multi‐Period Systematic Conservation Planning Reveals Certain Protection Gaps in Interprovincial Mountainous Areas

Given that the government manages nature reserves through administrative responsibility systems, this study adopted village‐level administrative boundaries as the basic planning units for SCP in order to clarify the relationship between biodiversity conservation and human development in Hubei Province under current and future conditions. This spatial scale is particularly effective in identifying the smallest conservation gaps within multi‐scale networks, and it provides a fundamental spatial framework for the coordinated management of protected areas across administrative borders (Li, Deng, et al. 2024; Li, Wang, et al. 2024). By coupling data from the SPP, HPP, and CPP, this study identified areas within the study region that are in urgent need of protection, aiming to fill the existing biodiversity conservation gaps (Xu and Li 2025). The integration of SCP simulations across multiple time periods revealed deeper structural deficiencies in the current reserve system (Zhou et al. 2002). Notably, the study uncovered 219 village‐level units with persistent conservation gaps located in transboundary mountainous areas, forming four prominent clusters: the source area of the JinQian River in Shiyan bordering Shaanxi, the Tongbai Mountain range in Suizhou bordering Henan, the Mufu Mountain belt in Xianning bordering Jiangxi, and the Wuling Mountain corridor in Enshi bordering Chongqing. This finding underscores two major concerns. First, it highlights potential shortcomings in China's current “national‐led, provincial‐municipal‐county‐coordinated” protected area governance framework (Gao et al. 2022). Areas along administrative boundaries may remain at risk of becoming management vacuums. Second, most existing protected areas have been delineated based on expert opinion and short‐term, static ecological data, often lacking systematic simulations that integrate multiple environmental factors. As a result, ecologically important areas with high biodiversity may have been overlooked (Li and Pimm 2020). Existing research indicates that mountainous and canyon regions along administrative borders, which experience less human disturbance, play critical ecological roles. For example, the upper reaches of the Jinqian River are a vital water conservation zone for the Han River, directly influencing the ecological integrity of the Danjiangkou Reservoir and the South‐to‐North Water Diversion Project. This area also supports the survival of rare and endangered species north of the Qinling Mountains. Similarly, the Tongbai, Mufu, and Wuling mountain systems serve as key climate refugia for species such as *Taxus wallichiana* var. *chinensis* and *Davidia involucrata* var. *vilmoriniana* (Chen et al. 2022; Zhao, Wang, et al. 2024). Incorporating these areas as priority conservation units into the natural reserve planning system would significantly enhance the overall quality and ecological connectivity of Hubei's conservation network. This “enhancement” is mainly reflected in two aspects: first, integrating unprotected areas of high conservation value into the system effectively fills the spatial and functional gaps in the existing conservation framework (Margules and Pressey 2000); second, by establishing ecological transition or buffer zones between existing protected areas, the connectivity and integrity among conservation patches are improved, thereby forming a more continuous and systematic conservation network (Saura et al. 2011). These improvements not only optimize the regional conservation pattern but also provide a more stable spatial foundation for the long‐term habitat sustainability of rare and endangered plant species. Designating and conserving these critical regions is not only essential for achieving the “30 by 30” global land protection target set in the Kunming‐Montreal Global Biodiversity Framework, but also provides theoretical and practical support for building a more comprehensive and tiered conservation planning system in China.

### 4.6 Limitations of the Study

This study, by coupling SPP, HPP, and CPP, simulated and quantitatively identified conservation gap areas within the study region, providing scientific recommendations for the improvement of China's national nature reserve system. However, some limitations remain. Regarding SPP, due to the limited availability of field survey data, this study only selected a subset of rare and endangered plant species with typical representativeness as target groups for biodiversity conservation. Other important taxonomic groups—such as birds, fish, and particularly data‐deficient endangered mammals—were not included in the assessment. Such a single‐taxon approach, especially one centered solely on plants, may fail to fully capture the broader conservation needs of regional biodiversity, thereby limiting the comprehensiveness of the results (Myers et al. 2000). Moreover, recent studies have shown that high‐resolution environmental data can significantly improve the understanding of species distribution drivers (Haesen et al. 2023). Due to limitations in the spatial resolution of climate, soil, and other datasets, this study used 1 km grid data for modeling species' potential distributions. Compared to finer‐resolution data, this spatial scale may lack sufficient detail to characterize microhabitat features accurately, which in turn may hinder the precision of protected area delineation and the development of targeted management strategies.

## 5 Conclusions

This study applied a combined SDMs–InVEST modeling approach to predict the current and future potential distributions, habitat quality, and carbon storage of 10 protected plant species in Hubei Province. By integrating multiple conservation perspectives and employing Marxan for simulation, the conservation gaps for these 10 species were identified. In the future, the overall carbon storage in Hubei Province shows an increasing trend, influenced by both urban expansion and forest restoration. The predictions from the combined models indicate that climate is the primary environmental variable affecting the potential distributions of the 10 protected plant species. In the future, the influence of human disturbance, EHPs, and habitat variables is expected to continue increasing, resulting in an overall contraction of 2.6% in the core potential geographic ranges of the 10 protected plant species. Furthermore, multi‐period simulations using Marxan identified persistent conservation gaps in the interprovincial mountainous areas of Hubei, forming four major clusters: the Jinqian River source area in Shiyan (bordering Shaanxi), the Tongbai Mountain corridor in Suizhou (bordering Henan), the Mufu Mountain range in Xianning, and the Wuling Mountain corridor in Enshi (bordering Chongqing). In the future, new nature reserves can be preferentially established from the 219 conservation gap planning units identified in this study, addressing the insufficient number and small area proportion of nature reserves in the northeastern and southeastern regions of Hubei Province. This study enhances the accuracy of identifying both biodiversity hotspots and high‐quality habitats for rare and endangered plants. It also contributes to improving the regional conservation planning system in China and provides scientific evidence and theoretical support for achieving the biodiversity targets set in the Kunming‐Montreal Global Biodiversity Framework.

## Author Contributions

**Dongyi Li:** conceptualization (lead), formal analysis (lead), methodology (lead), software (lead), validation (lead), visualization (lead), writing – original draft (lead). **Tingting Li:** investigation (equal), project administration (equal), resources (equal), supervision (equal), writing – review and editing (lead). **Juyang Wu:** formal analysis (equal), software (equal), validation (equal), writing – review and editing (equal). **Meng Zhang:** formal analysis (equal), software (equal), validation (equal). **Zhengxiang Wang:** conceptualization (lead), funding acquisition (lead), investigation (lead), project administration (lead), resources (lead), supervision (lead), writing – review and editing (lead).

## Funding

This work was supported by the National Natural Science Foundation of China, 42101065 and Knowledge Innovation Planning Project of Wuhan, China, 2023020201020424.

## Ethics Statement

The authors have nothing to report.

## Consent

The authors have nothing to report.

## Conflicts of Interest

The authors declare no conflicts of interest.

## Supporting information


Appendix S1.

## Data Availability

The data that supports the findings of this study are available in the Supporting Information of this article.

## References

[ece372672-bib-0001] Bedair, R. , A. A. Ibrahim , A. A. Alyamani , S. Aloufi , and S. Ramadan . 2021. “Impacts of Anthropogenic Disturbance on Vegetation Dynamics: A Case Study of Wadi Hagul, Eastern Desert, Egypt.” Plants 10: 1906. 10.3390/plants10091906. 34579436 PMC8466335

[ece372672-bib-0002] Bertuzzo, E. , F. Carrara , L. Mari , F. Altermatt , I. Rodriguez‐Iturbe , and A. Rinaldo . 2016. “Geomorphic Controls on Elevational Gradients of Species Richness.” Proceedings of the National Academy of Sciences of the United States of America 113: 1737–1742. 10.1073/pnas.1518922113. 26831107 PMC4763792

[ece372672-bib-0003] Bull, J. W. , K. B. Suttle , N. J. Singh , and E. Milner‐Gulland . 2013. “Conservation When Nothing Stands Still: Moving Targets and Biodiversity Offsets.” Frontiers in Ecology and the Environment 11: 203–210. 10.1890/120020.

[ece372672-bib-0004] Cabra‐Rivas, I. , A. Saldaña , P. Castro‐Díez , and L. Gallien . 2016. “A Multi‐Scale Approach to Identify Invasion Drivers and Invaders' Future Dynamics.” Biological Invasions 18: 411–426. 10.1007/s10530-015-1015-z.

[ece372672-bib-0005] Camps‐Valls, G. , M. Campos‐Taberner , Á. Moreno‐Martínez , et al. 2021. “A Unified Vegetation Index for Quantifying the Terrestrial Biosphere.” Science Advances 7: eabc7447. 10.1126/sciadv.abc7447. 33637524 PMC7909876

[ece372672-bib-0006] Chen, J. , S. Wang , and Y. Zou . 2022. “Construction of an Ecological Security Pattern Based on Ecosystem Sensitivity and the Importance of Ecological Services: A Case Study of the Guanzhong Plain Urban Agglomeration, China.” Ecological Indicators 136: 108688. 10.1016/j.ecolind.2022.108688.

[ece372672-bib-0007] Chuai, X. , X. Huang , L. Lai , W. Wang , J. Peng , and R. Zhao . 2013. “Land Use Structure Optimization Based on Carbon Storage in Several Regional Terrestrial Ecosystems Across China.” Environmental Science & Policy 25: 50–61. 10.1016/j.envsci.2012.05.005.

[ece372672-bib-0008] Chuai, X. , M. Xia , A. Xiang , L. Miao , R. Zhao , and T. Zuo . 2022. “Vegetation Coverage and Carbon Sequestration Changes in China's Forest Projects Area.” Global Ecology and Conservation 38: e02257. 10.1016/j.gecco.2022.e02257.

[ece372672-bib-0009] Cobos, M. E. , A. T. Peterson , N. Barve , and L. Osorio‐Olvera . 2019. “Kuenm: An R Package for Detailed Development of Ecological Niche Models Using Maxent.” PeerJ 7: e6281. 10.7717/peerj.6281. 30755826 PMC6368831

[ece372672-bib-0010] Cunha‐Zeri, G. , J. F. Guidolini , E. A. Branco , and J. P. Ometto . 2022. “How Sustainable Is the Nitrogen Management in Brazil? A Sustainability Assessment Using the Entropy Weight Method.” Journal of Environmental Management 316: 115330. 10.1016/j.jenvman.2022.115330. 35658265

[ece372672-bib-0011] Dai, Y. , H. Huang , Y. Qing , J. Li , and D. Li . 2023. “Ecological Response of an Umbrella Species to Changing Climate and Land Use: Habitat Conservation for Asiatic Black Bear in the Sichuan‐Chongqing Region, Southwestern China.” Ecology and Evolution 13: e10222. 10.1002/ece3.10222. 37384242 PMC10293704

[ece372672-bib-0098] DNRHP . 2025. “Geographic Location and Land Resources of Hubei Province.”

[ece372672-bib-0012] Dobson, A. P. , A. D. Bradshaw , and A. J. Baker . 1997. “Hopes for the Future: Restoration Ecology and Conservation Biology.” Science 277: 515–522. 10.1016/j.envc.2025.101182.

[ece372672-bib-0013] Dubuis, A. , S. Giovanettina , L. Pellissier , J. Pottier , P. Vittoz , and A. Guisan . 2013. “Improving the Prediction of Plant Species Distribution and Community Composition by Adding Edaphic to Topo‐Climatic Variables.” Journal of Vegetation Science 24: 593–606. 10.1111/jvs.12002.

[ece372672-bib-0014] Dutta, T. , M. De Barba , N. Selva , et al. 2023. “An Objective Approach to Select Surrogate Species for Connectivity Conservation.” Frontiers in Ecology and Evolution 11: 1078649. 10.3389/fevo.2023.1078649.

[ece372672-bib-0015] Elith, J. , S. J. Phillips , T. Hastie , M. Dudík , Y. E. Chee , and C. J. Yates . 2011. “A Statistical Explanation of MaxEnt for Ecologists.” Diversity and Distributions 17: 43–57. 10.1111/j.1472-4642.2010.00725.x.

[ece372672-bib-0016] Fujiwara, K. 1987. “Aims and Methods of Phytosociology or “Vegetation Science”.” Plant Ecology and Taxonomy to the Memory of Dr. Satoshi Nakanishi.

[ece372672-bib-0017] Gao, C. , Z. Fan , C. Ma , J. Yang , and S. Guo . 2024. “Prediction of Suitable Area of *Camellia reticulata* Under Climate Change Based on the Biomod2 Ensemble Model.” Chinese Journal of Ecology 43: 3526. 10.13292/j.1000-4890.202411.020.

[ece372672-bib-0018] Gao, H. , Y. Weng , Y. Lu , and Y. Du . 2022. “An Innovative Framework on Spatial Boundary Optimization of Multiple International Designated Land Use.” Sustainability 14: 587. 10.3390/su14020587.

[ece372672-bib-0021] Guo, Y. , Z. Zhao , H. Qiao , et al. 2020. “Challenges and Development Trend of Species Distribution Model.” Advances in Earth Science 35: 1292–1305. 10.11867/j.issn.1001-8166.2020.110.

[ece372672-bib-0022] Haddad, N. M. , L. A. Brudvig , J. Clobert , et al. 2015. “Habitat Fragmentation and Its Lasting Impact on Earth's Ecosystems.” Science Advances 1: e1500052. 10.1126/sciadv.1500052. 26601154 PMC4643828

[ece372672-bib-0023] Haesen, S. , J. Lenoir , E. Gril , et al. 2023. “Microclimate Reveals the True Thermal Niche of Forest Plant Species.” Ecology Letters 26: 2043–2055. 10.1111/ele.14312. 37788337

[ece372672-bib-0099] HPFB . 2025. “Plant and Animal Resources.”

[ece372672-bib-0024] Huang, J. , B. Chen , C. Liu , J. Lai , J. Zhang , and K. Ma . 2012. “Identifying Hotspots of Endemic Woody Seed Plant Diversity in China.” Diversity and Distributions 18: 673–688. 10.1111/j.1472-4642.2011.00845.x.

[ece372672-bib-0025] Huang, R. , H. Du , Y. Wen , et al. 2022. “Predicting the Distribution of Suitable Habitat of the Poisonous Weed *Astragalus variabilis* in China Under Current and Future Climate Conditions.” Frontiers in Plant Science 13: 921310. 10.3389/fpls.2022.921310. 36204071 PMC9531759

[ece372672-bib-0026] Ke, X. , and L. Tang . 2019. “Impact of Cascading Processes of Urban Expansion and Cropland Reclamation on the Ecosystem of a Carbon Storage Service in Hubei Province, China.” Acta Ecologica Sinica 39: 672–683. 10.5846/stxb201712042177.

[ece372672-bib-0027] Li, B. V. , and S. L. Pimm . 2020. “How China Expanded Its Protected Areas to Conserve Biodiversity.” Current Biology 30: R1334–R1340. 10.1016/j.cub.2020.09.025. 33202224

[ece372672-bib-0028] Li, D. , T. Li , W. Xue , Y. Xia , and Z. Wang . 2025. “Prediction and Analysis of Potential Habitat Distribution of *Taxus wallichiana var. chinensis* Under Climate Change: A Case Study of Hubei Province.” Ecology and Environmental Sciences 34: 1398. 10.16258/j.cnki.1674-5906.2025.09.007.

[ece372672-bib-0029] Li, J. , C. Deng , G. Duan , Z. Wang , Y. Zhang , and G. Fan . 2024. “Potentially Suitable Habitats of *Daodi* Goji Berry in China Under Climate Change.” Frontiers in Plant Science 14: 1279019. 10.3389/fpls.2023.1279019. 38264027 PMC10803630

[ece372672-bib-0030] Li, J. , S. Dong , Y. Li , Y. Wang , Z. Li , and F. Li . 2022. “Effects of Land Use Change on Ecosystem Services in the China–Mongolia–Russia Economic Corridor.” Journal of Cleaner Production 360: 132175. 10.1016/j.jclepro.2022.132175.

[ece372672-bib-0031] Li, X. , Z. Wang , S. Wang , and Z. Qian . 2024. “MaxEnt and Marxan Modeling to Predict the Potential Habitat and Priority Planting Areas of *Coffea arabica* in Yunnan, China Under Climate Change Scenario.” Frontiers in Plant Science 15: 1471653. 10.3389/fpls.2024.1471653. 39670274 PMC11635998

[ece372672-bib-0032] Li, Y. , W. Shao , and J. Jiang . 2022. “Predicting the Potential Global Distribution of *Sapindus mukorossi* Under Climate Change Based on MaxEnt Modelling.” Environmental Science and Pollution Research 29: 21751–21768. 10.1007/s11356-021-17294-9. 34773237

[ece372672-bib-0033] Liang, X. , Q. Guan , K. C. Clarke , S. Liu , B. Wang , and Y. Yao . 2021. “Understanding the Drivers of Sustainable Land Expansion Using a Patch‐Generating Land Use Simulation (PLUS) Model: A Case Study in Wuhan, China.” Computers, Environment and Urban Systems 85: 101569. 10.1016/j.compenvurbsys.2020.101569.

[ece372672-bib-0034] Long, T. , J. Tang , N. W. Pilfold , X. Zhao , and T. Dong . 2021. “Predicting Range Shifts of Davidia Involucrata Ball. Under Future Climate Change.” Ecology and Evolution 11: 12779–12789. 10.1002/ece3.8023. 34594538 PMC8462142

[ece372672-bib-0035] Luedtke, J. A. , J. Chanson , K. Neam , et al. 2023. “Ongoing Declines for the World's Amphibians in the Face of Emerging Threats.” Nature 622: 308–314. 10.1038/s41586-023-06578-4. 37794184 PMC10567568

[ece372672-bib-0036] Ma, Y. , J. Li , W. Cao , C. Yin , and L. Huan . 2024. “Projecting the Carbon Sink Potential and Contribution of Grain for Green Program in the Beijing‐Tianjin‐Hebei Region.” Acta Geographica Sinica 79: 732–746. 10.11821/dlxb202403011.

[ece372672-bib-0037] Mao, D. , Z. Wang , J. Wu , et al. 2018. “China's Wetlands Loss to Urban Expansion.” Land Degradation and Development 29: 2644–2657. 10.1002/ldr.2939.

[ece372672-bib-0038] Margules, C. R. , and R. L. Pressey . 2000. “Systematic Conservation Planning.” Nature 405: 243–253. 10.1038/35012251. 10821285

[ece372672-bib-0039] Marques, A. , I. S. Martins , T. Kastner , et al. 2019. “Increasing Impacts of Land Use on Biodiversity and Carbon Sequestration Driven by Population and Economic Growth.” Nature Ecology & Evolution 3: 628–637. 10.1038/s41559-019-0824-3. 30833755 PMC6443044

[ece372672-bib-0040] Meilleur, B. A. , and T. Hodgkin . 2004. “In Situ Conservation of Crop Wild Relatives: Status and Trends.” Biodiversity and Conservation 13: 663–684. 10.1023/b:bioc.0000011719.03230.17.

[ece372672-bib-0041] Mo, L. , C. M. Zohner , P. B. Reich , et al. 2023. “Integrated Global Assessment of the Natural Forest Carbon Potential.” Nature 624: 92–101. 10.1038/s41586-023-06723-z. 37957399 PMC10700142

[ece372672-bib-0042] Müllers, Y. , J. A. Postma , H. Poorter , et al. 2022. “Shallow Roots of Different Crops Have Greater Water Uptake Rates Per Unit Length Than Deep Roots in Well‐Watered Soil.” Plant and Soil 481: 475–493. 10.1007/s11104-022-05650-8.

[ece372672-bib-0043] Murakami, D. , T. Yoshida , and Y. Yamagata . 2021. “Gridded GDP Projections Compatible With the Five SSPs (Shared Socioeconomic Pathways).” Frontiers in Built Environment 7: 760306. 10.3389/fbuil.2021.760306.

[ece372672-bib-0044] Myers, N. , R. A. Mittermeier , C. G. Mittermeier , G. A. Da Fonseca , and J. Kent . 2000. “Biodiversity Hotspots for Conservation Priorities.” Nature 403: 853–858. 10.1038/35002501. 10706275

[ece372672-bib-0045] Naiman, R. J. , and H. Décamps . 1997. “The Ecology of Interfaces: Riparian Zones.” Annual Review of Ecology and Systematics 28: 621–658. 10.1146/annurev.ecolsys.28.1.621.

[ece372672-bib-0046] Olds, A. D. , R. M. Connolly , K. A. Pitt , P. S. Maxwell , S. Aswani , and S. Albert . 2014. “Incorporating Surrogate Species and Seascape Connectivity to Improve Marine Conservation Outcomes.” Conservation Biology 28: 982–991. 10.1111/cobi.12242. 24527964

[ece372672-bib-0047] Pacifici, M. , W. B. Foden , P. Visconti , et al. 2015. “Assessing Species Vulnerability to Climate Change.” Nature Climate Change 5: 215–224. 10.1038/nclimate2448.

[ece372672-bib-0048] Pang, S. E. , J. F. Slik , R. A. Chisholm , and E. L. Webb . 2024. “Conserving Southeast Asian Trees Requires Mitigating Both Climate and Land‐Use Change.” Nature Sustainability 7: 1313–1323. 10.1038/s41893-024-01417-4.

[ece372672-bib-0049] Parmesan, C. , and G. Yohe . 2003. “A Globally Coherent Fingerprint of Climate Change Impacts Across Natural Systems.” Nature 421: 37–42. 10.1038/nature01286. 12511946

[ece372672-bib-0050] Pecl, G. T. , M. B. Araújo , J. D. Bell , et al. 2017. “Biodiversity Redistribution Under Climate Change: Impacts on Ecosystems and Human Well‐Being.” Science 355: eaai9214. 10.1126/science.aai9214. 28360268

[ece372672-bib-0051] Phillips, S. J. , R. P. Anderson , and R. E. Schapire . 2006. “Maximum Entropy Modeling of Species Geographic Distributions.” Ecological Modelling 190: 231–259. 10.1016/j.ecolmodel.2005.03.026.

[ece372672-bib-0052] Phillips, S. J. , and M. Dudík . 2008. “Modeling of Species Distributions With Maxent: New Extensions and a Comprehensive Evaluation.” Ecography 31: 161–175. 10.1111/j.0906-7590.2008.5203.x.

[ece372672-bib-0053] Qi, X. , C. Chen , H. P. Comes , et al. 2012. “Molecular Data and Ecological Niche Modelling Reveal a Highly Dynamic Evolutionary History of the East Asian Tertiary Relict *Cercidiphyllum* (Cercidiphyllaceae).” New Phytologist 196: 617–630. 10.1111/j.1469-8137.2012.04242.x. 22845876

[ece372672-bib-0054] Ralimanana, H. , A. L. Perrigo , R. J. Smith , et al. 2022. “Madagascar's Extraordinary Biodiversity: Threats and Opportunities.” Science 378. 10.1126/science.adf1466. 36454830

[ece372672-bib-0055] Rawat, D. S. , A. S. Bagri , M. Parveen , M. Nautiyal , P. Tiwari , and J. K. Tiwari . 2021. “Pattern of Species Richness and Floristic Spectrum Along the Elevation Gradient: A Case Study From Western Himalaya, India.” Acta Ecologica Sinica 41: 545–551. 10.1016/j.chnaes.2021.03.012.

[ece372672-bib-0056] Riva, F. , C. J. Martin , C. Galán Acedo , et al. 2024. “Incorporating Effects of Habitat Patches Into Species Distribution Models.” Journal of Ecology 112: 2162–2182. 10.1111/1365-2745.14403.

[ece372672-bib-0057] Sallustio, L. , A. De Toni , A. Strollo , et al. 2017. “Assessing Habitat Quality in Relation to the Spatial Distribution of Protected Areas in Italy.” Journal of Environmental Management 201: 129–137. 10.1016/j.jenvman.2017.06.031. 28651222

[ece372672-bib-0058] Saura, S. , C. Estreguil , C. Mouton , and M. Rodríguez‐Freire . 2011. “Network Analysis to Assess Landscape Connectivity Trends: Application to European Forests (1990–2000).” Ecological Indicators 11: 407–416. 10.1016/j.ecolind.2010.06.011.

[ece372672-bib-0059] Senior, R. A. , R. Bagwyn , D. Leng , A. K. Killion , W. Jetz , and D. S. Wilcove . 2024. “Global Shortfalls in Documented Actions to Conserve Biodiversity.” Nature 630: 387–391. 10.1038/s41586-024-07498-7. 38839953 PMC11168922

[ece372672-bib-0060] Shafer, S. L. , P. J. Bartlein , and R. S. Thompson . 2001. “Potential Changes in the Distributions of Western North America Tree and Shrub Taxa Under Future Climate Scenarios.” Ecosystems 4: 200–215. 10.1007/s10021-001-0004-5.

[ece372672-bib-0061] Shin, Y. , G. F. Midgley , E. R. M. Archer , et al. 2022. “Actions to Halt Biodiversity Loss Generally Benefit the Climate.” Global Change Biology 28: 2846–2874. 10.1111/gcb.16109. 35098619 PMC9303674

[ece372672-bib-0062] Soto‐Navarro, C. , C. Ravilious , A. Arnell , et al. 2020. “Mapping Co‐Benefits for Carbon Storage and Biodiversity to Inform Conservation Policy and Action.” Philosophical Transactions of the Royal Society B 375: 20190128. 10.1098/rstb.2019.0128. PMC7017768 31983334

[ece372672-bib-0063] Street, G. M. 2020. “Habitat Suitability and Distribution Models With Applications in R.” Journal of Wildlife Management 84, no. 6. 1212–1213.

[ece372672-bib-0064] Suzuki, N. , and K. L. Parker . 2019. “Proactive Conservation of High‐Value Habitat for Woodland Caribou and Grizzly Bears in the Boreal Zone of British Columbia, Canada.” Biological Conservation 230: 91–103. 10.1016/j.biocon.2018.12.013.

[ece372672-bib-0065] Tang, J. , H. Lu , Y. Xue , et al. 2021. “Data‐Driven Planning Adjustments of the Functional Zoning of Houhe National Nature Reserve.” Global Ecology and Conservation 29: e01708. 10.1016/j.gecco.2021.e01708.

[ece372672-bib-0066] Thuiller, W. , B. Lafourcade , R. Engler , and M. B. Araújo . 2009. “BIOMOD – A Platform for Ensemble Forecasting of Species Distributions.” Ecography 32: 369–373. 10.1111/j.1600-0587.2008.05742.x.

[ece372672-bib-0067] Tian, C. , J. Zhong , Q. You , et al. 2025. “Land Use Modeling and Habitat Quality Assessment Under Climate Scenarios: A Case Study of the Poyang Lake Basin.” Ecological Indicators 172: 113292. 10.1016/j.ecolind.2025.113292.

[ece372672-bib-0068] UNEP‐WCMC, IUCN, NGS . 2020. “Protected Planet Live Report 2020.”

[ece372672-bib-0069] Valavi, R. , G. Guillera‐Arroita , J. J. Lahoz‐Monfort , and J. Elith . 2022. “Predictive Performance of Presence‐Only Species Distribution Models: A Benchmark Study With Reproducible Code.” Ecological Monographs 92, no. 1: e01486.

[ece372672-bib-0070] Van Proosdij, A. S. J. , M. S. M. Sosef , J. J. Wieringa , and N. Raes . 2016. “Minimum Required Number of Specimen Records to Develop Accurate Species Distribution Models.” Ecography 39: 542–552. 10.1111/ecog.01509.

[ece372672-bib-0071] Vignali, S. , A. G. Barras , R. Arlettaz , and V. Braunisch . 2020. “ *SDMtune*: An R Package to Tune and Evaluate Species Distribution Models.” Ecology and Evolution 10: 11488–11506. 10.1002/ece3.6786. 33144979 PMC7593178

[ece372672-bib-0072] Wang, L. , J. Gao , W. Shen , Y. Shi , and H. Zhang . 2021. “Carbon Storage in Vegetation and Soil in Chinese Ecosystems Estimated by Carbon Transfer Rate Method.” Ecosphere 12: e03341. 10.1002/ecs2.3341.

[ece372672-bib-0073] Wang, Q. , J. Ge , W. Li , Z. Zhang , F. Shen , and X. Xu . 2010. “Establishment of Nature Reserve in Hubei Province: Status Quo and Gap Analysis.” Environmental Science & Technology 33: 190–195. 10.3969/j.issn.1003-6504.2010.04.045.

[ece372672-bib-0074] Wang, X. , X. Meng , and Y. Long . 2022. “Projecting 1 Km‐Grid Population Distributions From 2020 to 2100 Globally Under Shared Socioeconomic Pathways.” Scientific Data 9: 563. 10.1038/s41597-022-01675-x. 36097271 PMC9466344

[ece372672-bib-0075] Watson, J. E. , N. Dudley , D. B. Segan , and M. Hockings . 2014. “The Performance and Potential of Protected Areas.” Nature 515: 67–73. 10.1038/nature13947. 25373676

[ece372672-bib-0076] Watts, M. E. , I. R. Ball , R. S. Stewart , et al. 2009. “Marxan With Zones: Software for Optimal Conservation Based Land‐and Sea‐Use Zoning.” Environmental Modelling & Software 24: 1513–1521. 10.1016/j.envsoft.2009.06.005.

[ece372672-bib-0077] Wei, Z. , L. Ling , Q. Wang , and D. Luo . 2025. “Multi‐Scenario Land Use Change Dynamic Simulation and Carbon Stock Assessment of Man–Nature in Border Mountainous Areas.” Sustainability 17: 1695. 10.3390/su17041695.

[ece372672-bib-0078] Xu, M. , L. Niu , X. Wang , and Z. Zhang . 2023. “Evolution of Farmland Landscape Fragmentation and Its Driving Factors in the Beijing‐Tianjin‐Hebei Region.” Journal of Cleaner Production 418: 138031. 10.1016/j.jclepro.2023.138031.

[ece372672-bib-0079] Xu, Y. , and C. Li . 2025. “A Novel Multidimensional Framework for Bridging Conservation Gaps and Optimizing the System of Natural Reserves for Biodiversity: A Case Study of Dongying, China.” Ecological Indicators 170: 113088. 10.1016/j.ecolind.2025.113088.

[ece372672-bib-0082] Yang, H. , H. Zhang , Y. Wang , et al. 2025. “Urban Bird Diversity Conservation Plan Based on the MaxEnt Model and InVEST Model: A Case Study of Jinan, China.” Ecological Indicators 174: 113463. 10.1016/j.ecolind.2025.113463.

[ece372672-bib-0083] Yang, Q. , T. Li , Z. Wang , Y. Xu , H. Zhang , and L. Li . 2019. “Spatial Scale Analysis of the Species Diversity and Distribution of Rare and Endangered Plants in Northwest Hubei, China.” Plant Science Journal 37: 464–473. 10.11913/PSJ.2095-0837.2019.40464.

[ece372672-bib-0084] Ye, C. , H. Liu , H. Qin , J. Shu , Z. Zhou , and X. Jin . 2023. “Geographical Distribution and Conservation Strategy of National Key Protected Wild Plants of China.” iScience 26: 107364. 10.1016/j.isci.2023.107364. 37539030 PMC10393829

[ece372672-bib-0085] Yuan, R. , N. Zhang , and Q. Zhang . 2024. “The Impact of Habitat Loss and Fragmentation on Biodiversity in Global Protected Areas.” Science of the Total Environment 931: 173004. 10.1016/j.scitotenv.2024.173004. 38710390

[ece372672-bib-0086] Zeng, Q. , X. Ye , Y. Cao , X. Chuai , and H. Xu . 2023. “Impact of Expanded Built‐Up Land on Ecosystem Service Value by Considering Regional Interactions.” Ecological Indicators 153: 110397. 10.1016/j.ecolind.2023.110397.

[ece372672-bib-0087] Zhang, C. , H. Tian , G. Chen , et al. 2012. “Impacts of Urbanization on Carbon Balance in Terrestrial Ecosystems of the Southern United States.” Environmental Pollution 164: 89–101. 10.1016/j.envpol.2012.01.020. 22343525

[ece372672-bib-0088] Zhang, F. , J. Zhan , Q. Zhang , L. Yao , and W. Liu . 2017. “Impacts of Land Use/Cover Change on Terrestrial Carbon Stocks in Uganda.” Physics and Chemistry of the Earth, Parts A/B/C 101: 195–203. 10.1016/j.pce.2017.03.005.

[ece372672-bib-0089] Zhang, H. , J. M. Chase , and J. Liao . 2024. “Habitat Amount Modulates Biodiversity Responses to Fragmentation.” Nature Ecology & Evolution 8: 1437–1447. 10.1038/s41559-024-02445-1. 38914711

[ece372672-bib-0090] Zhang, H. , J. Luo , J. Wu , and H. Dong . 2024. “Dynamic Response of Carbon Storage to Future Land Use/Land Cover Changes Motivated by Policy Effects and Core Driving Factors.” Journal of Plant Ecology 17: rtae042. 10.1093/jpe/rtae042.

[ece372672-bib-0092] Zhang, Y. , Z. Mei , and X. Zheng . 2023. Temporal and Spatial Variation and Prediction of Carbon Storage in the Yellow River Delta Coupled With InVEST and MCE‐CA‐Markov Model, 6. Chinese Society of Landscape Architecture. 10.26914/c.cnkihy.2023.002299.

[ece372672-bib-0093] Zhang, Y. , J. She , X. Long , and M. Zhang . 2022. “Spatio‐Temporal Evolution and Driving Factors of Eco‐Environmental Quality Based on RSEI in Chang‐Zhu‐Tan Metropolitan Circle, Central China.” Ecological Indicators 144: 109436. 10.1016/j.ecolind.2022.109436.

[ece372672-bib-0094] Zhao, L. , J. Li , R. L. Barrett , et al. 2024. “Spatial Heterogeneity of Extinction Risk for Flowering Plants in China.” Nature Communications 15: 6352. 10.1038/s41467-024-50704-3. PMC11284212 39069525

[ece372672-bib-0095] Zhao, P. , L. Wang , Y. Huang , et al. 2024. “Comprehensive Evaluation and Scenario Simulation for Determining the Optimal Conservation Priority of Ecological Services in Danjiangkou Reservoir Area, China.” Ecological Indicators 169: 112906. 10.1016/j.ecolind.2024.112906.

[ece372672-bib-0096] Zhou, C. H. , C. F. Lee , J. Li , and Z. W. Xu . 2002. “On the Spatial Relationship Between Landslides and Causative Factors on Lantau Island, Hong Kong.” Geomorphology 43: 197–207. 10.1016/S0169-555X(01)00130-1.

[ece372672-bib-0097] Zhu, L. , R. Song , S. Sun , Y. Li , and K. Hu . 2022. “Land Use/Land Cover Change and Its Impact on Ecosystem Carbon Storage in Coastal Areas of China From 1980 to 2050.” Ecological Indicators 142: 109178. 10.1016/j.ecolind.2022.109178.

