# Supplementary material for: Potential Distribution Modeling and Conservation Gap Identification for Rare and Endangered Plant Species: A Case Study of 10 Species in Hubei Province
Source: Ecol Evol. 2025 Dec 18;15(12):e72672. doi: 10.1002/ece3.72672 (PMC12712545; doi:10.1002/ece3.72672)
Supplement: Supplementary file 1 — Appendix S1. [file ECE3-15-e72672-s001.docx]

**Appendix Supplementary data**

**The specific formula for habitat quality is as follows：**

$Q_{xj}=H_{j}\left[1-\left(\frac{D_{xj}^{z}}{D_{xj}^{z}+k^{z}}\right)\right]$ (A.1)

In this equation, *Q_xj_*​ represents the habitat quality of grid cell x*x* under land use type *j*. It is determined jointly by *H_j_* (the habitat suitability of land use type *j*) and *D_xj_* (the threat level to grid cell *x* under land use type *j*). The parameter *k* is the half-saturation constant, typically set to half the maximum value of *D_xj_*, and *z* is a constant scaling factor, taking a value of 2.5. The specific formula for calculating *D_xj_* is as follows:

$D_{xj}=\sum_{r=1}^{R} \sum_{y=1}^{Y_{r}} \left(\frac{W_{r}}{\sum_{r=1}^{R} W_{r}}\right)r_{y}i_{rxy}\beta_{x}S_{jr}$ (A.2)

In this equation, *r* denotes a threat factor and *R* represents the total number of threat factors; *y* indicates the total grid cells for threat factor *r*; *Y_r_*​ denotes the set of threat grid cells for all threat factors; *W_r_* is the weight of threat factor *r* (0 ≤*W_r_* ≤ 1); *r_y_* serves as a threat grid indicator (binary); *i_rxy_*​ quantifies the threat level from threat grid *y* to grid cell *x*; *β_x_* denotes the accessibility of grid cell *x* (0 ≤*β_x_* ≤ 1); and *S_jr_* represents the sensitivity of land use type *j* to threat factor *r* (0 ≤*S_jr_* ≤ 1).

**Probability-Weighted Average Method: Specific Formula：**

$P_{EMwmean}=\sum_{i=1}^{N} \omega_{i}\times P_{i}$ (A.3)

In this equation, *P_EMwmean_*​ represents the final predicted probability of the ensemble model; *P_i_*_​_ denotes the predicted probability of the *i*-th individual model; *ω_i_*_​_ is the weight assigned to the *i*-th individual model; and *N* signifies the total number of individual models. Additionally, the constraint $\sum_{i=1}^{N} \omega_{i}=1$ is imposed to ensure the weights sum to unity. The specific formula for calculating *ω_i_*_​_ is as follows:

$\omega_{i}=\frac{S_{i}}{\sum_{j=1}^{N} S_{i}}$ (A.4)

In this equation, *ω_i_*_​_ denotes the weight assigned to the *i*-th individual model; *S_i_*_​_ is the True Skill Statistic (TSS) evaluation score of the *i*-th model; and *N* represents the number of individual models with TSS scores exceeding 0.8.

**Specific Formula for the Comprehensive Landscape Fragmentation Index (CLFI):**

Data normalization was first performed to address significant heterogeneity in measurement units and value ranges across indicators. Min-Max normalization was applied to eliminate dimensional effects(Yan et al., 2021), as expressed by the following formula:

$X=\frac{X-X_{min}}{X_{max}-X_{min}}$ (A.5)

where *X_min_​* denotes the global minimum value of the indicator across the entire dataset, and *X_max_* represents its global maximum value.

The values of NP and PD exhibit a *positive* correlation with the degree of fragmentation; thus, their normalized results are directly retained. Conversely, AREA_MN and AI show a *negative* correlation with fragmentation degree, requiring mathematical inversion through the following formula:

$X_{adjusted }=1-X_{norm }$ (A.6)

where *X_adjusted_*​ denotes the mathematically inverted values of AREA_MN and AI, and *X_norm_*​ represents the normalized values of these indicators, respectively.

Finally, the equal weighting method (where each indicator's weight *w*=1/4​) was applied to balance the contributions of all indicators. The CLFI is then computed as follows:

$LIF=\frac{NP_{norm}+PD_{norm}+AREA\_MN_{adjusted }+AI_{adjusted }}{4}$ (A.7)

This index ranges from 0 to 1, with higher values indicating a greater degree of landscape fragmentation.

**Here's the professional translation of the requested content:**

The first step in the entropy weight method involves normalization (Yan et al., 2021). Since the factors in this study—GDP and land use data—constitute positive-value indicators, they are processed using the following formula:

$HDI_{ij}=\frac{X_{j}-X_{min\left(j\right)}}{X_{max\left(j\right)}-X_{min\left(j\right)}}$ (A.8)

The second step of the entropy weight method determines the entropy value:

$V_{j}=-K^{-1}\sum_{j=1}^{n} U_{ij}\ln U_{ij}$ (A.9)

$K=\frac{1}{\ln\left(m\right)}$ (A.10)

$U_{ij}=\frac{HDI_{ij}}{\sum_{i=1}^{n} HDI_{ij}}$ (A.11)

Finally, the entropy weight of each indicator is calculated as follows, which serves as the weight *W_j​_* for each disturbance factor:

$W_{j}=\frac{1-V_{j}}{\sum_{j=1}^{n} \left(1-V_{j}\right)}$ (A.12)

where *X_max(j)_*​ denotes the maximum value of indicator *j*; *X_min(j)_​* represents the minimum value of indicator *j*; *X_j​_*indicates the original value of indicator *j*; *HDI_ij​_* signifies the normalized result of indicator *j*; *V_j​_* is the entropy value of indicator *j*; *W_j​_*denotes the entropy weight of indicator *j*; *n* specifies the number of indicators; and *m* defines the number of samples.

**Supplementary tables.**

Table S1. Ten plant species collected by this institute and their conservation status.

| **Family** | **Genus** | **Species** | **Protection level** | | | **Number of distribution points** | | |
| --- | --- | --- | --- | --- | --- | --- | --- | --- |
|  |  |  | **NKPWP** | **IUCN** | **CITES** | **Investigation** | **Online** | **Total** |
| Taxaceae | Taxus | *Taxus wallichiana* var*. chinensis* | Ⅰ | VU | Ⅱ | 49 | 24 | 73 |
| Fabaceae | Glycine | *Glycine soja* | Ⅱ | NE | - | 46 | 23 | 69 |
| Actinidiaceae | Actinidia | *Actinidia chinensis* | Ⅱ | NE | - | 76 | 38 | 113 |
| Nyssaceae | Davidia | *Davidia involucrata* var. *vilmoriniana* | Ⅰ | VU | - | 33 | 16 | 49 |
| Berberidaceae | Dysosma | *Dysosma versipellis* | Ⅱ | VU | - | 26 | 13 | 39 |
| Cercidiphyllaceae | Cercidiphyllum | *Cercidiphyllum japonicum* | Ⅱ | LC | - | 26 | 13 | 39 |
| Rubiaceae | Emmenopterys | *Emmenopterys henryi* | Ⅱ | NE | - | 41 | 20 | 61 |
| Polygonaceae | Fagopyrum | *Fagopyrum dibotrys* | Ⅱ | LC | - | 22 | 11 | 33 |
| Taxaceae | Torreya | *Torreya fargesii* | Ⅱ | VU | - | 42 | 21 | 63 |
| Trochodendraceae | Tetracentron | *Tetracentron sinense* | Ⅱ | NE | Ⅲ | 27 | 13 | 40 |

Note: 1. List of National Key Protected Wild Plants(NKPWP): Ⅰ-Class I nationally protected plants, Ⅱ-Class II nationally protected plants; 2. IUCN Red List of Threatened Species(IUCN): VU – Vulnerable, NE – Not Evaluated, LC – Least Concern; 3. Convention on International Trade in Endangered Species of Wild Fauna and Flora (CITES): Ⅱ–Appendix II, Ⅲ–Appendix III.

Table S2. Driving factors of land use change.

| **Data types** | **Data Name** | **Spatial resolution** | **Is future data available** | **Sources of the data** |
| --- | --- | --- | --- | --- |
| Climate | Annual Mean Temperature (BIO1) | 30 arc-seconds | √ | WorldClim (https://www.worldclim.org/) |
|  | Mean Diurnal Range (BIO2) |  |  |  |
|  | Isothermality (BIO3) |  |  |  |
|  | Temperature Seasonality (BIO4) |  |  |  |
|  | Max Temperature of Warmest Month (BIO5) |  |  |  |
|  | Min Temperature of Coldest Month (BIO6) |  |  |  |
|  | Temperature Annual Range (BIO7) |  |  |  |
|  | Mean Temperature of Wettest Quarter (BIO8) |  |  |  |
|  | Mean Temperature of Driest Quarter (BIO9) |  |  |  |
|  | Mean Temperature of Warmest Quarter (BIO10) |  |  |  |
|  | Mean Temperature of Coldest Quarter (BIO11) |  |  |  |
|  | Annual Precipitation (BIO12) |  |  |  |
|  | Precipitation of Wettest Month (BIO13) |  |  |  |
|  | Precipitation of Driest Month (BIO14) |  |  |  |
|  | Precipitation Seasonality (BIO15) |  |  |  |
|  | Precipitation of Wettest Quarter (BIO16) |  |  |  |
|  | Precipitation of Driest Quarter (BIO17) |  |  |  |
|  | Precipitation of Warmest Quarter (BIO18) |  |  |  |
|  | Precipitation of Coldest Quarter (BIO19) |  |  |  |
| Habitat | Elevation | 30m | - | Earthdata Search (https://search.earthdata.nasa.gov/) |
|  | Slope |  |  |  |
|  | Aspect |  |  |  |
|  | Soil (AWC for Rootable Soil Depth) | 1km | - | Food and Agriculture Organization of the United Nations (https://gaez.fao.org/) |
|  | Soil (Electric Conductivity) |  |  |  |
|  | Soil (Organic Carbon Content) |  |  |  |
|  | Soil (PH in water) |  |  |  |
|  | Soil (Sand) |  |  |  |
|  | Soil (Silt) |  |  |  |
|  | Soil (Texture class) |  |  |  |
|  | Soil (Soil Unit Symbo) |  |  |  |
|  | Kernel Normalized Difference Vegetation Index | 30m | - | National Science and Technology Resource Sharing Service Platform (https://www.escience.org.cn/) |
| Land Cover/Land Use | Land Use Type | 30m | √ | Resource and Environmental Science and Data Center (https://www.resdc.cn/) |
|  | Patch Metrics (Four Indicators) |  |  |  |
|  | Landscape Metrics (Sixteen Indicators) |  |  |  |
| Anthropogenic Disturbance | Gridded GDP | 1km | √ | Gridded GDP Projections Compatible With the Five SSPs (Shared Socioeconomic Pathways) (https://doi.org/10.3389/fbuil.2021.760306/) |
|  | 1 km-grid population | 1km | √ | Projecting 1 km-grid population distributions from 2020 to 2100 globally under shared socioeconomic pathways (https://doi.org/10.1038/s41597-022-01675-x/) |
|  | Road network density(Railway) | 1km | - | National Catalogue Service for Geographic Information(https://www.webmap.cn/) |
|  | Road network density(Highways) |  |  |  |
|  | Road network density(Class I Roads) |  |  |  |
|  | Road network density(Class Ⅱ Roads) |  |  |  |
|  | Road network density(Class Ⅲ Roads) |  |  |  |
|  | Road network density (Class Ⅳ Roads) |  |  |  |
|  | Road network density(Subgrade Roads) |  |  |  |

Note: For detailed indicator names of Patch Metrics and Landscape Metrics, refer to Table S6.

Table S3. The driving factors of land use models.

| **Species Name** | **Type of Environmental Factor** | | | | | | | | **Total** |
| --- | --- | --- | --- | --- | --- | --- | --- | --- | --- |
|  | **Climate** | **Total** | **Habitat** | **Total** | **Land Use** | **Total** | **Anthropogenic Disturbance** | **Total** |  |
| *Taxus wallichiana* var*. chinensis* | BIO6, BIO8 | 2 | kNDVI, Slope, WRB_PHA | 3 | LUCC, CA 5 | 2 | RND Highways, RND Railway, RND Class I Roads, RND Class Ⅱ Roads | 4 | 11 |
| *Glycine soja* | BIO6, BIO7 | 2 | kNDVI, Slope, WRB_PHA | 3 | LUCC, FRAC, NP 4 | 3 | RND Class I Roads, RND Class Ⅱ Roads, RND Class Ⅳ Roads, GDP, Population | 5 | 13 |
| *Actinidia chinensis* | BIO4, BIO11 | 2 | Sand, Slope, WRB_PHA, Silt, Texture | 5 | LUCC, FRAC_MN, NP 5 | 3 | RND Highways, RND Railway, RND Class Ⅱ Roads, RND Class Ⅲ Roads, RND Class Ⅳ Roads, Population | 6 | 16 |
| *Davidia involucrata* var. *vilmoriniana* | BIO10 | 1 | kNDVI, PH in water, WRB_PHA, Texture | 4 | NP 1 | 1 | RND Highways, RND Class I Roads, RND Class Ⅱ Roads, GDP, Population | 5 | 11 |
| *Dysosma versipellis* | BIO1 | 1 | kNDVI, Texture, WRB_PHA | 3 | CA 1, SHDI | 2 | RND Highways, RND Railway, RND Class Ⅲ Roads, Population | 4 | 10 |
| *Cercidiphyllum japonicum* | BIO4, BIO11 | 2 | kNDVI, AWC | 2 | LUCC, TE | 2 | RND Railway, RND Class Ⅱ Roads, GDP | 3 | 9 |
| *Emmenopterys henryi* | BIO4 | 1 | EC, Texture, WRB_PHA | 3 | CA 2, NP 3 | 2 | RND Highways, RND Railway, RND Class I Roads, GDP, Population | 5 | 11 |
| *Fagopyrum dibotrys* | BIO7 | 1 | kNDVI, EC, WRB_PHA | 3 | LUCC, FRAC, CA 2, CA 3 | 4 | RND Railway | 1 | 9 |
| *Torreya fargesii* | BIO4 | 1 | kNDVI, Slope, WRB_PHA, Aspect | 4 | LUCC, TE, CA 2, CA 3 | 4 | RND Highways, RND Railway, RND Class I Roads, Population | 4 | 13 |
| *Tetracentron sinense* | BIO4 | 1 | kNDVI, WRB_PHA | 2 | FRAC_MN, CA 2 | 2 | RND Highways, RND Railway, RND Class Ⅲ Roads, GDP | 4 | 9 |

Note: BIO1 = Annual Mean Temperature; BIO4 = Temperature Seasonality; BIO6 = Min Temperature of Coldest Month; BIO7 = Temperature Annual Range; BIO8 = Mean Temperature of Wettest Quarter; BIO10 = Mean Temperature of Warmest Quarter; BIO11 = Mean Temperature of Coldest Quarter; kNDVI = Kernel Normalized Difference Vegetation Index; WRB_PHA = Soil classification (FAO WRB 2022 standard); Texture = Texture class; AWC = Available water capacity (for rootable soil depth); EC = Electric conductivity; LUCC = Land use and cover change; CA 1, 2, 3, 5 = area of land-use types: farmland, forest, grassland, and built-up land, respectively; NP 1, 3, 4, 5 = number of patches of farmland, grassland, water bodies, and built-up land, respectively; FRAC = Fractal Dimension; FRAC_MN = Landscape Fractal Dimension (mean fractal dimension); SHDI = Shannon’s Diversity Index; TE = Total Edge; RND Highways, RND Railway, RND I–IV = densities of highways, railways, and Class I–IV roads, respectively; GDP = gridded GDP; Population = population at 1-km grid resolution.

Table S4. The driving factors of land use models.

| **Data types** | **Data Name** | **Sources of the data** | **Unit** |
| --- | --- | --- | --- |
| Natural Environmental Factors | Elevation | Earthdata Search (https://search.earthdata.nasa.gov/) | m |
|  | Slope |  | ° |
|  | Aspect |  | - |
|  | Distance to Water Bodies | Resource and Environmental Science and Data Center (https://www.resdc.cn/) | m |
| Socioeconomic Factors | Population Distribution | Projecting 1 km-grid population distributions from 2020 to 2100 globally under shared socioeconomic pathways (https://doi.org/10.1038/s41597-022-01675-x/) | people/km2 |
|  | GDP Distribution | Gridded GDP Projections Compatible With the Five SSPs (Shared Socioeconomic Pathways) (https://doi.org/10.3389/fbuil.2021.760306/) | 1000,000 USD/km² |
|  | Distance to Urban Land | Resource and Environmental Science and Data Center (https://www.resdc.cn/) | m |
|  | Distance to Rural Settlements |  | m |
|  | Distance to Railway | National Catalogue Service for Geographic Information(https://www.webmap.cn/) | m |
|  | Distance to Highways |  | m |
|  | Distance to Class I Roads |  | m |
|  | Distance to Class Ⅱ Roads |  | m |
|  | Distance to Class Ⅲ Roads |  | m |
|  | Distance to Class Ⅳ Roads |  | m |
|  | Distance to Subgrade Roads |  | m |

Table S5. Threat factor parameter.

| **Threat** | **Decay** | **Max_Dist** | **Weight** |
| --- | --- | --- | --- |
| Cultivated | linear | 4 | 0.6 |
| Construction | exponential | 7 | 1 |
| Class I Roads | linear | 1 | 0.4 |
| Class Ⅱ Roads | linear | 0.5 | 0.4 |
| Highways | linear | 2 | 0.6 |
| Railway | linear | 3 | 0.8 |

Note: 1. Decay describes how a threat’s influence declines with distance from its source; InVEST supports two decay types: "linear" or "exponential"; 2. Max_Dist specifies the maximum distance (typically in meters) over which the threat affects habitat; 3. Weight indicates the relative severity/impact of the threat compared with other threats (typically ranged between 0 and 1).

Table S6. Sensitivity parameter.

| **Types** | **Habitat** | **Cultivated** | **Construction** | **Class I Roads** | **Class Ⅱ Roads** | **Highways** | **Railway** |
| --- | --- | --- | --- | --- | --- | --- | --- |
| Cultivated | 0.4 | 0 | 0.5 | 0.5 | 0.5 | 0.6 | 0.6 |
| Forest | 1 | 0.3 | 0.1 | 0.3 | 0.3 | 0.4 | 0.4 |
| Grassland | 1 | 0.3 | 0.2 | 0.3 | 0.3 | 0.4 | 0.4 |
| Water | 1 | 0.1 | 0 | 0 | 0 | 0 | 0 |
| Construction | 0 | 0 | 0 | 0.6 | 0.6 | 0.7 | 0.7 |
| Unusedland | 0 | 0 | 0 | 0 | 0 | 0 | 0 |

Table S7. Carbon density of different land-use types in Hubei Province.

| **Land Use Type** | **Aboveground Biomass** | **Belowground Biomass** | **Dead Biomass** | **Soil** | **References** |
| --- | --- | --- | --- | --- | --- |
| Cultivated | 16.49 | 10.89 | 2.11 | 75.82 | Zhang et al., 2017; Ke and Tang, 2019 |
| Forest | 30.14 | 6.03 | 2.78 | 100.15 | Chuai et al., 2013; Ke and Tang, 2019 |
| Grassland | 14.29 | 17.15 | 7.28 | 87.05 | Chuai et al., 2013; Ke and Tang, 2019 |
| Water | 0 | 0 | 0 | 0 | Zhang et al., 2012; Ke and Tang, 2019 |
| Construction | 7.61 | 1.52 | 0 | 34.33 | Zhang et al., 2017; Ke and Tang, 2019 |
| Unused land | 10.36 | 2.07 | 0.96 | 34.42 | Zhang et al., 2017; Ke and Tang, 2019 |

Note: 1. the units for aboveground biomass, belowground biomass, dead organic matter, and soil organic matter are t/ha (tons per hectare; equivalent to t·hm⁻² ).

Table S8. EHPs indicators and their names.

| **Landscape Metrics** | **Indicator Abbreviations** | **Patch Metrics** | **Indicator Abbreviations** |
| --- | --- | --- | --- |
| Class Area | CA(CA1-6) | Patch Size | AREA |
| Landscape Fractal Dimension | FRAC_MN | Core Area | CORE |
| Number of Patches | NP(NP Total, NP1-6) | Edge Length | PERIM |
| Total Edge | TE | Fractal Dimension | FRAC |
| Shannon’s Diversity Index | SHDI | - | - |

Note: 1. CA1-6 represent the class areas of cultivated, forest, grassland, water, construction, and unusedland, respectively; 2. NP Total and NP1-6 denote the total number of patches and the number of patches for cultivated, forest, grassland, water, construction, and unusedland, respectively.

Table S9. The setting of Targets and SPF values is based on the protection levels defined by IUCN, NKPWP, and CITES.

| **Protected Target Name** | **Protected Plant Diversity Hotspot Zone** | | **Species–Habitat Priority Zone** | | **Species–Carbon Synergy Zone** | | **Habitat–Carbon Synergy Zone** | | **Other Areas** | |
| --- | --- | --- | --- | --- | --- | --- | --- | --- | --- | --- |
|  | **Target** | **SPF** | **Target** | **SPF** | **Target** | **SPF** | **Target** | **SPF** | **Target** | **SPF** |
| *Taxus wallichiana* var. *chinensis* | 0.8 | 100 | 0.7 | 90 | 0.7 | 90 | - | - | 0.6 | 80 |
| *Glycine soja* | 0.5 | 100 | 0.4 | 90 | 0.4 | 90 | - | - | 0.3 | 80 |
| *Actinidia chinensis* | 0.5 | 100 | 0.4 | 90 | 0.4 | 90 | - | - | 0.3 | 80 |
| *Davidia involucrata* var*. vilmoriniana* | 0.8 | 100 | 0.7 | 90 | 0.7 | 90 | - | - | 0.6 | 80 |
| *Dysosma versipellis* | 0.8 | 100 | 0.7 | 90 | 0.7 | 90 | - | - | 0.6 | 80 |
| *Cercidiphyllum japonicum* | 0.5 | 100 | 0.4 | 90 | 0.4 | 90 | - | - | 0.3 | 80 |
| *Emmenopterys henryi* | 0.5 | 100 | 0.4 | 90 | 0.4 | 90 | - | - | 0.3 | 80 |
| *Fagopyrum dibotrys* | 0.5 | 100 | 0.4 | 90 | 0.4 | 90 | - | - | 0.3 | 80 |
| *Torreya fargesii* | 0.8 | 100 | 0.7 | 90 | 0.7 | 90 | - | - | 0.6 | 80 |
| *Tetracentron sinense* | 0.5 | 100 | 0.4 | 90 | 0.4 | 90 | - | - | 0.3 | 80 |
| High Carbon Stock Zone | 0.3 | 50 | - | - | 0.2 | 40 | 0.2 | 40 | 0.1 | 30 |
| High Value Habitat Zone | 0.3 | 50 | 0.2 | 40 | - | - | 0.2 | 40 | 0.1 | 30 |

Table S10. Optimization results of MaxEnt modeling parameters for different plant species.

| **Species Name** | **RM Value** | **FC Type** |
| --- | --- | --- |
| *Taxus wallichiana* var. *chinensis* | 0.5 | l |
| *Glycine soja* | 1.6 | lpt |
| *Actinidia chinensis* | 2.1 | lt |
| *Davidia involucrata* var. *vilmoriniana* | 3.7 | t |
| *Dysosma versipellis* | 1.4 | p |
| *Cercidiphyllum japonicum* | 0.2 | lp |
| *Emmenopterys henryi* | 2.9 | qt |
| *Fagopyrum dibotrys* | 2.2 | lt |
| *Torreya fargesii* | 2.9 | lt |
| *Tetracentron sinense* | 1.6 | lt |

Note: 1. RM Value = regularization multiplier (dimensionless); 2. FC Type = feature class (feature types used by MaxEnt).

Table S11. Accuracy assessment of ensemble model simulations for ten plant species.

| **Species name** | **AUC** | **TSS** | **Kappa** |
| --- | --- | --- | --- |
| *Taxus wallichiana* var. *chinensis* | 0.989 | 0.922 | 0.660 |
| *Glycine soja* | 0.962 | 0.853 | 0.412 |
| *Actinidia chinensis* | 0.988 | 0.905 | 0.73 |
| *Davidia involucrata* var. *vilmoriniana* | 0.990 | 0.947 | 0.673 |
| *Dysosma versipellis* | 0.990 | 0.961 | 0.615 |
| *Cercidiphyllum japonicum* | 0.987 | 0.940 | 0.573 |
| *Emmenopterys henryi* | 0.980 | 0.903 | 0.586 |
| *Fagopyrum dibotrys* | 0.999 | 0.984 | 0.922 |
| *Torreya fargesii* | 0.982 | 0.907 | 0.643 |
| *Tetracentron sinense* | 0.989 | 0.949 | 0.585 |
| Mean value | 0.986 | 0.927 | 0.640 |

Table S12. Contribution values of environmental factors to ten plant species and their projected future changes.

|  | **Current environmental factors' contribution values to potentially suitable habitats** | | | | **The percentage change in future environmental factors' contribution to potentially suitable habitats** | | | |
| --- | --- | --- | --- | --- | --- | --- | --- | --- |
| **Species Name** | **Climate** | **EHPs** | **Habitat** | **Human Disturbance** | **Climate** | **EHPs** | **Habitat** | **Human Disturbance** |
| *Taxus wallichiana* var. *chinensis* | 40.09% | 0.30% | 33.25% | 26.36% | -7.96% | 2.28% | 6.42% | -0.75% |
| *Glycine soja* | 46.46% | 2.30% | 29.35% | 21.89% | -7.38% | -0.81% | 13.35% | -5.16% |
| *Actinidia chinensis* | 37.18% | 5.58% | 23.17% | 34.06% | -13.64% | 3.39% | 26.60% | -16.35% |
| *Davidia involucrata* var. *vilmoriniana* | 71.99% | 14.67% | 4.33% | 9.01% | 7.01% | -12.15% | 1.06% | 4.07% |
| *Dysosma versipellis* | 24.28% | 4.63% | 8.47% | 62.62% | -2.95% | -0.69% | -1.38% | 5.01% |
| *Cercidiphyllum japonicum* | 56.99% | 2.35% | 18.03% | 22.63% | -12.74% | 17.19% | -13.28% | 8.83% |
| *Emmenopterys henryi* | 8.33% | 43.13% | 1.54% | 47.00% | 2.35% | -24.62% | 0.63% | 21.65% |
| *Fagopyrum dibotrys* | 13.84% | 46.26% | 35.71% | 4.19% | -0.70% | 24.12% | -25.64% | 2.21% |
| *Torreya fargesii* | 3.40% | 16.72% | 69.92% | 9.96% | 5.07% | 4.02% | -10.44% | 1.35% |
| *Tetracentron sinense* | 29.84% | 0.37% | 39.14% | 30.65% | -1.56% | 3.82% | -3.88% | 1.62% |
| Mean value | 34.03% | 13.24% | 27.11% | 25.63% | -2.76% | 1.07% | 0.19% | 1.49% |

Table S13. Current and future potential distribution areas of ten plant species and their changes.

|  | **Current Potential Suitable Habitat Area(km^2^)** | | | | | **Future Potential Suitable Habitat Change(%)** | | |
| --- | --- | --- | --- | --- | --- | --- | --- | --- |
| **Species Name** | | **High Suitability** | **Moderate Suitability** | **Low Suitability** | **Total** | **Expasion** | **Loss** | **Net Change** |
| *Taxus wallichiana* var. *chinensis* | | 1279 | 3017 | 4062 | 8358 | 44.80 | 8.81 | 35.99 |
| *Glycine soja* | | 2681 | 4470 | 4325 | 11476 | 128.42 | 1.69 | 126.73 |
| *Actinidia chinensis* | | 2889 | 6175 | 7783 | 16847 | 3.45 | 44.56 | -41.11 |
| *Davidia involucrata* var. *vilmoriniana* | | 1579 | 2956 | 3475 | 8010 | 21.96 | 14.46 | 7.50 |
| *Dysosma versipellis* | | 1465 | 2059 | 2421 | 5945 | 12.46 | 12.51 | -0.05 |
| *Cercidiphyllum japonicum* | | 1920 | 3488 | 4942 | 10350 | 39.96 | 2.49 | 37.47 |
| *Emmenopterys henryi* | | 2628 | 4894 | 7759 | 15281 | 26.87 | 9.87 | 17.00 |
| *Fagopyrum dibotrys* | | 4954 | 7222 | 8889 | 21065 | 6.04 | 46.54 | -40.50 |
| *Torreya fargesii* | | 4455 | 7274 | 9335 | 21064 | 1.37 | 48.33 | -46.96 |
| *Tetracentron sinense* | | 2217 | 3375 | 5161 | 10753 | 0.33 | 27.95 | -27.61 |


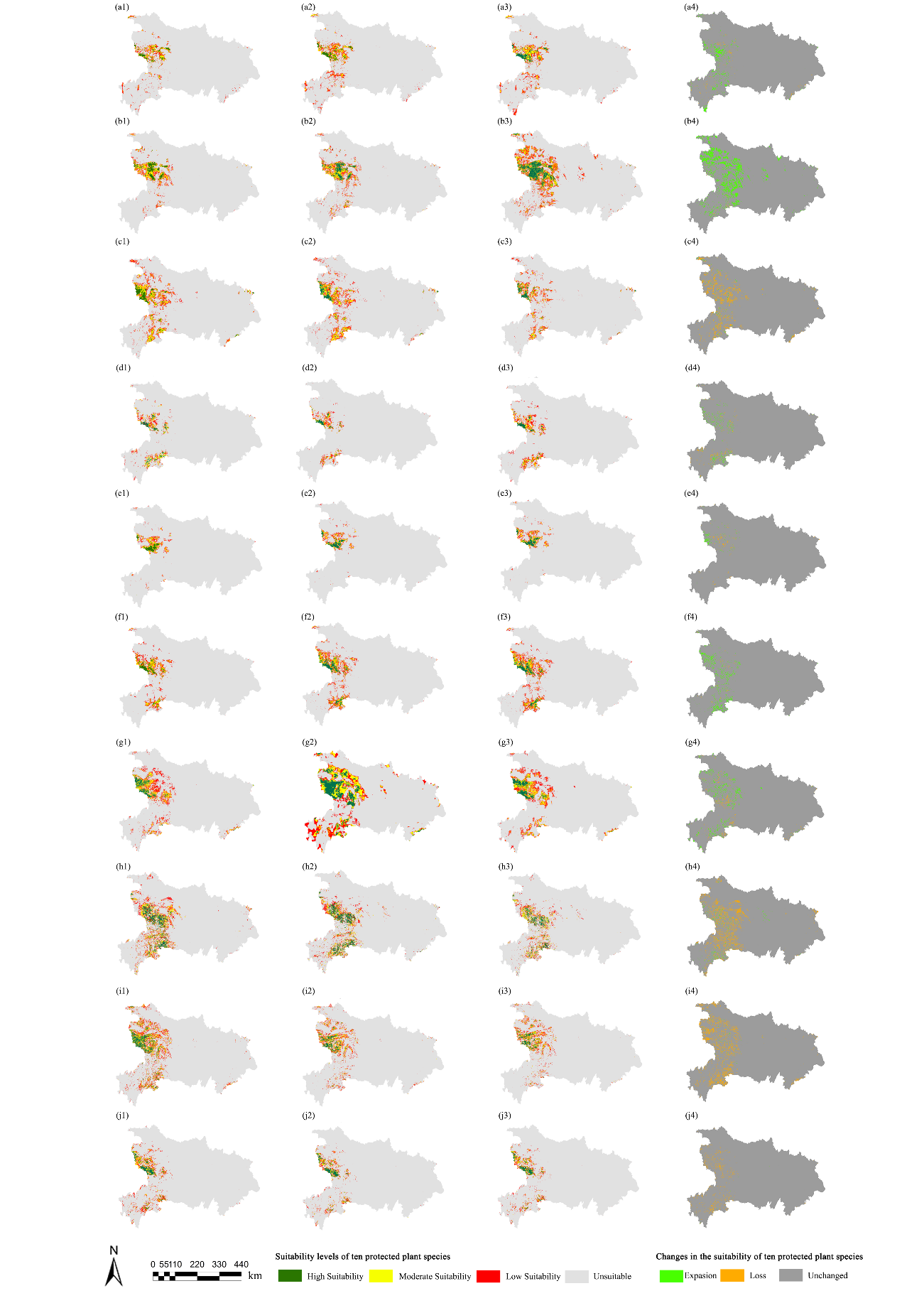
**Supplementary figures.**

Fig. S1. Current and projected potential geographic distribution patterns for ten conservation-priority plant species (a–j denote the protected plant species *Taxus wallichiana* var. *chinensis*, *Glycine soja*, *Actinidia chinensis*, *Davidia involucrata* var. *vilmoriniana*, *Dysosma versipellis*, *Cercidiphyllum japonicum*, *Emmenopterys henryi*, *Fagopyrum dibotrys*, *Torreya fargesii*, and *Tetracentron sinense*, respectively; 1–4 denote the species’ potential distribution in 2020, 2030, and 2060, and the change in potential distribution between 2020 and 2060, respectively).


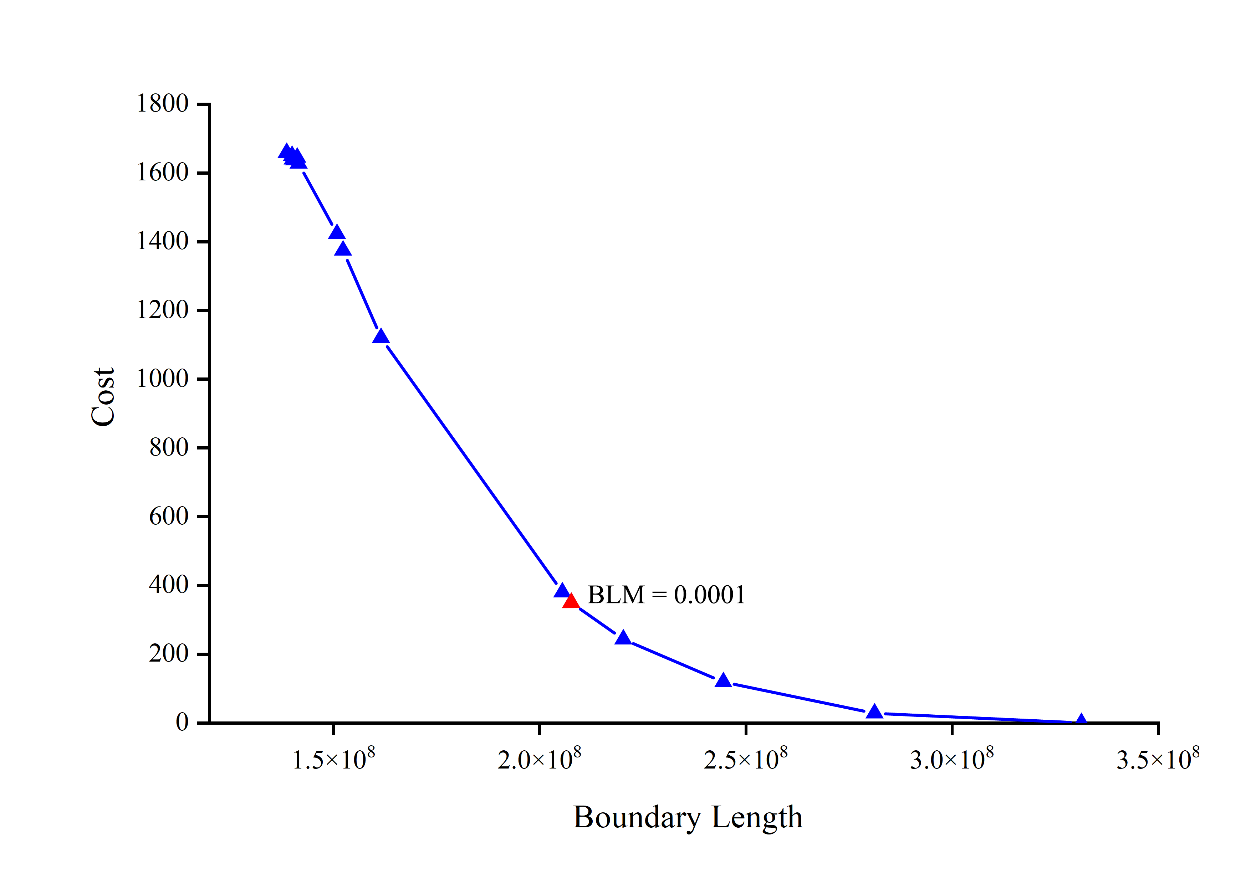


Fig. S2. Optimization results of the BLM parameter in Marxan modelling(When BLM = 0.0001, a solution can be obtained that has a low cost value and a minimized boundary length).


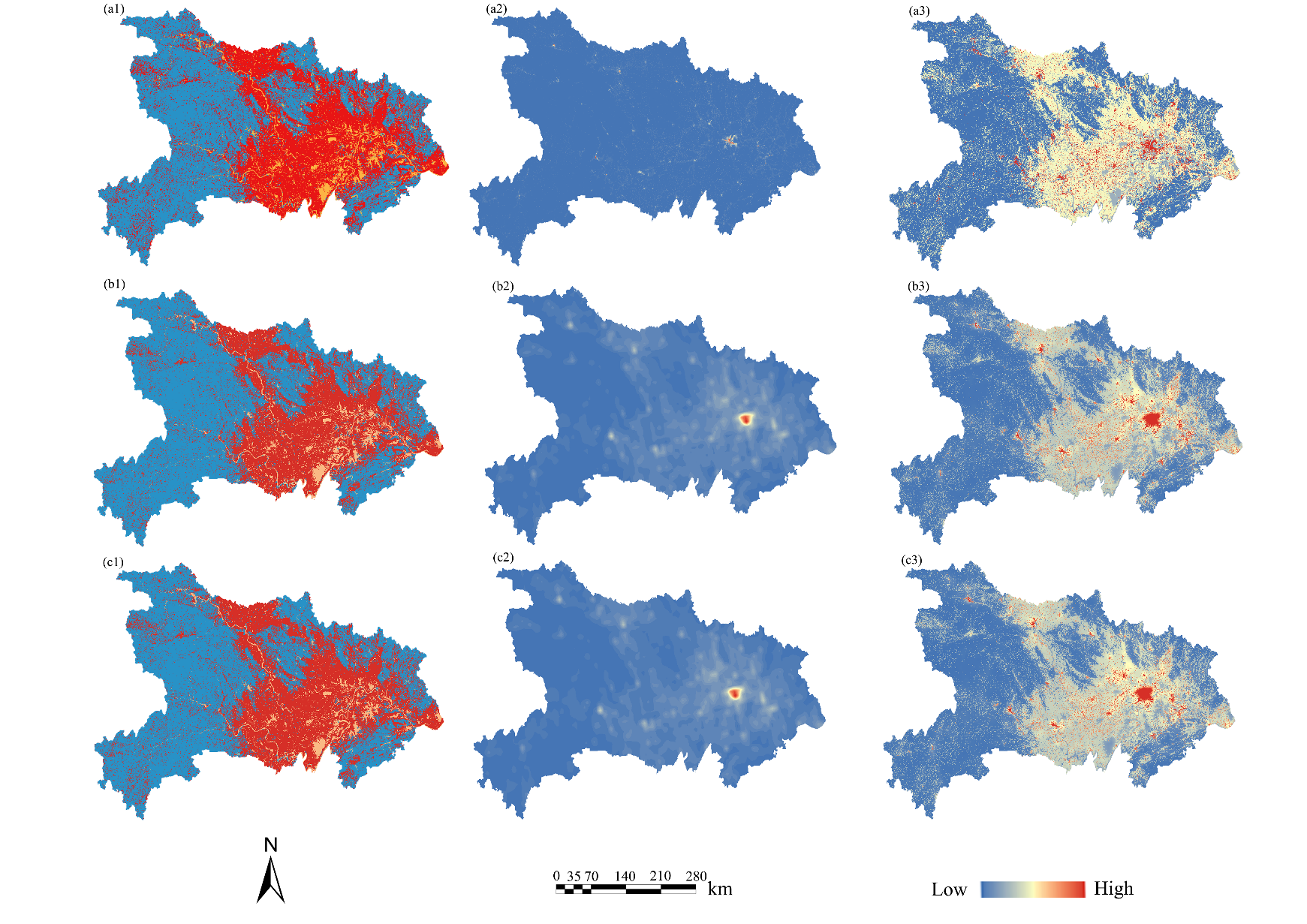


Fig. S3. Changes in current and future HDI distribution patterns (a. HDI 2020, b. HDI 2030, c. HDI 2060; 1-3 respectively representing land use and GDP data weighted by the entropy method).


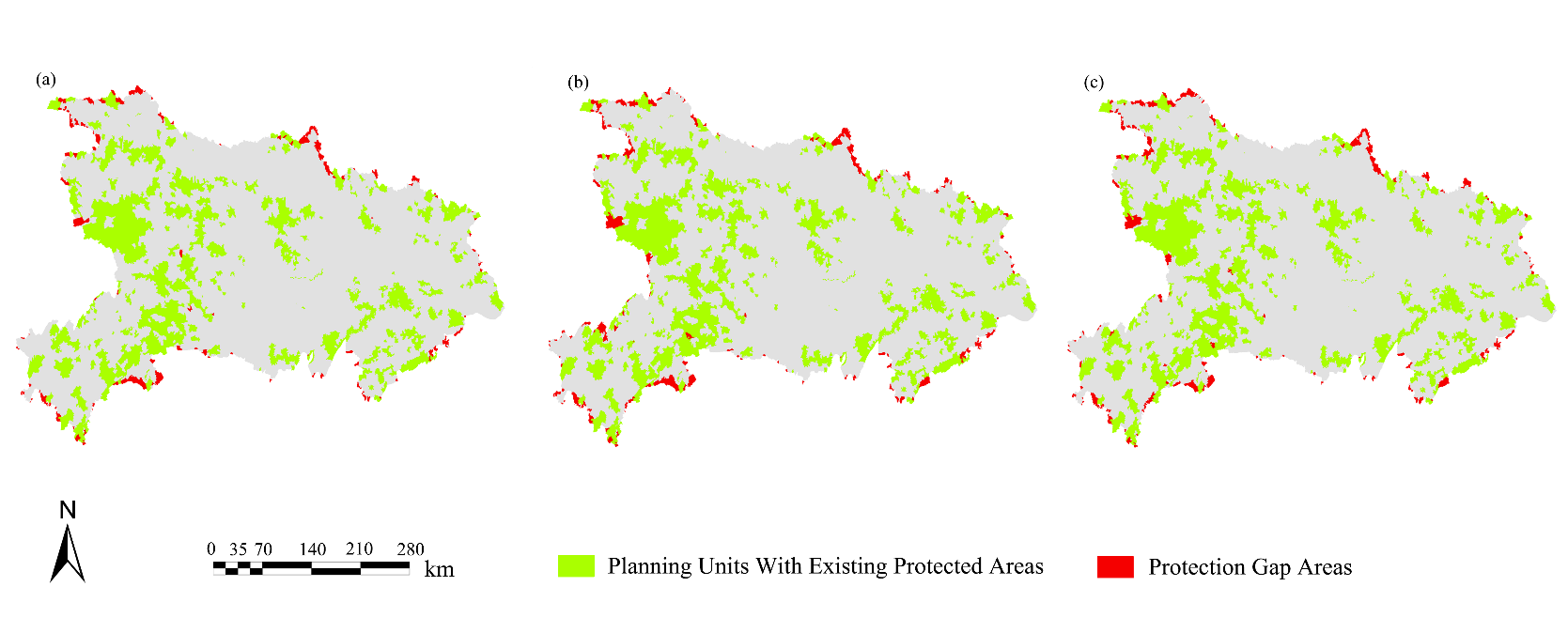


Fig. S4. Conservation gap areas identified in Hubei Province across temporal phases (a. 2020, b. 2030, c. 2060).

***References***

Yan, B., Y. Li, Y. Qin, J. Yan, and W. Shi. 2021. “Spatial-Temporal Analysis of the Comparative Advantages of Dairy Farming: Taking 18 Provinces or Municipalities in China as an Example.” *Computers and Electronics in Agriculture* 180: 105846. https://doi.org/10.1016/j.compag.2020.105846.
